# Cation Diffusion‐Mediated Displaced Reaction‐Transformation in Green Steelmaking

**DOI:** 10.1002/advs.76583

**Published:** 2026-07-16

**Authors:** Guangyi Guo, Paul Paciok, Baptiste Bienvenu, Longqi Bai, Barak Ratzker, Marc Heggen, Xuyang Zhou, Dierk Raabe

**Affiliations:** ^1^ Max Planck Institute for Sustainable Materials Düsseldorf Germany; ^2^ Ernst Ruska‐Centre for Microscopy and Spectroscopy with Electrons, Jülich Forschungszentrum Jülich Germany; ^3^ Center for Advancing Materials Performance from the Nanoscale (CAMP‐Nano) State Key Laboratory for Mechanical Behavior of Materials Xi'an Jiaotong University Xi'an China

**Keywords:** cation diffusion, hydrogen‐based direct reduction, in situ transmission electron microscopy, magnetite

## Abstract

Hydrogen‐based direct reduction of iron oxides shows great potential for decarbonizing the iron and steelmaking industry. However, deciphering such complex, multistage reactions remains challenging due to the concurrent occurrence of multiple nonlinearly coupled processes. Here, the in‐operando reduction dynamics of iron oxide ranging from 200 °C–1000 °C is revealed using time‐resolved hydrogen‐environmental scanning transmission electron microscopy (with a hydrogen gas pressure of 3 Pa) combined with bulk analysis and atomistic modeling. Proposing a general cation‐mediated redox mechanism in Fe_3_O_4_, cation diffusion triggers a spatially displaced reaction–transformation and drives the ensuing dynamic phase transformations and reduction steps that extend significantly beyond the reaction front. At 727 °C the spatial displacement is 2–4 orders of magnitude above the previously assumed reaction‐front thickness. The results underscore the cross‐scale universality and pivotal role of cation diffusion in enabling rapid hydrogen‐based reduction of metal oxides, offering not only fundamental atomic‐scale insights to help render the steelmaking industry more sustainable, but also a better understanding of related processes in batteries and corrosion.

## Graphical abstract

The removal of O lattice atoms by environmental H_2_ at the surface of Fe_3_O_4_ results in Fe enrichment, driving the inward diffusion of Fe cations into the bulk. Through hopping between unoccupied and initially occupied sites, cation diffusion triggers a spatially displaced reaction‐transformation and drives subsequent dynamic phase transformations and reduction steps that extend significantly beyond the reaction front.

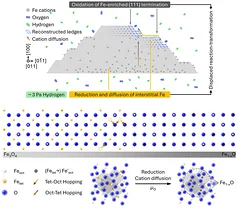

## 1 Introduction

Ironmaking history essentially unfolds how we thermodynamically compete with O for extracting Fe from its feedstock, ${\mathrm{Fe}}_{2}O_{3}$ (hematite), ${\mathrm{Fe}}_{3}O_{4}$ (magnetite) and ${\mathrm{Fe}}_{1-x}O$, (wüstite, where x denotes the degree of Fe deficiency). Carbon‐carrying fossil reductants are still extensively used to date in the iron and steel industry, rendering it a high ${\mathrm{CO}}_{2}$ footprint contributor with approximately 8 % of global annual carbon emissions in the past few years [1, 2, 3]. Hydrogen‐based direct reduction of iron oxides emerges as promising, scalable technology to decarbonize the steel sector [4, 5, 6, 7], for instance by replacing the blast‐furnace pathway, the biggest single industrial process driving global warming, which alone accounts for over 70 % of global steel production [1, 8]. While the kinetics of iron oxide reduction have been extensively investigated, the local solid‐state transport processes that connect the gas‐solid reduction with the actual movement of the transformation front remains less clearly resolved. Understanding and theoretically describing such complex, multistage reactions remains challenging due to the concurrent occurrence of multiple nonlinearly coupled processes involving mass transport, surface topology reconstruction, phase transformations, and interface migration [9, 10, 11, 12, 13, 14].

The interplay among gas access, surface reaction rates, and bulk ion transport (ion diffusion) determines both the microstructure and kinetics of hydrogen‐based direct reduction (HyDR) of metal oxides [15, 16, 17, 18]. As schematically plotted in Figure 1a, the reaction and ion diffusion‐mediated transformation rates reach a rate balance at the crossover point. When migration of Fe cation interstitials or vacancies proceeds faster than the local reduction reaction (reaction‐limited), the phase transformation sites can decouple from the initial reduction front—a process we term a displaced reaction–transformation.

**FIGURE 1:**
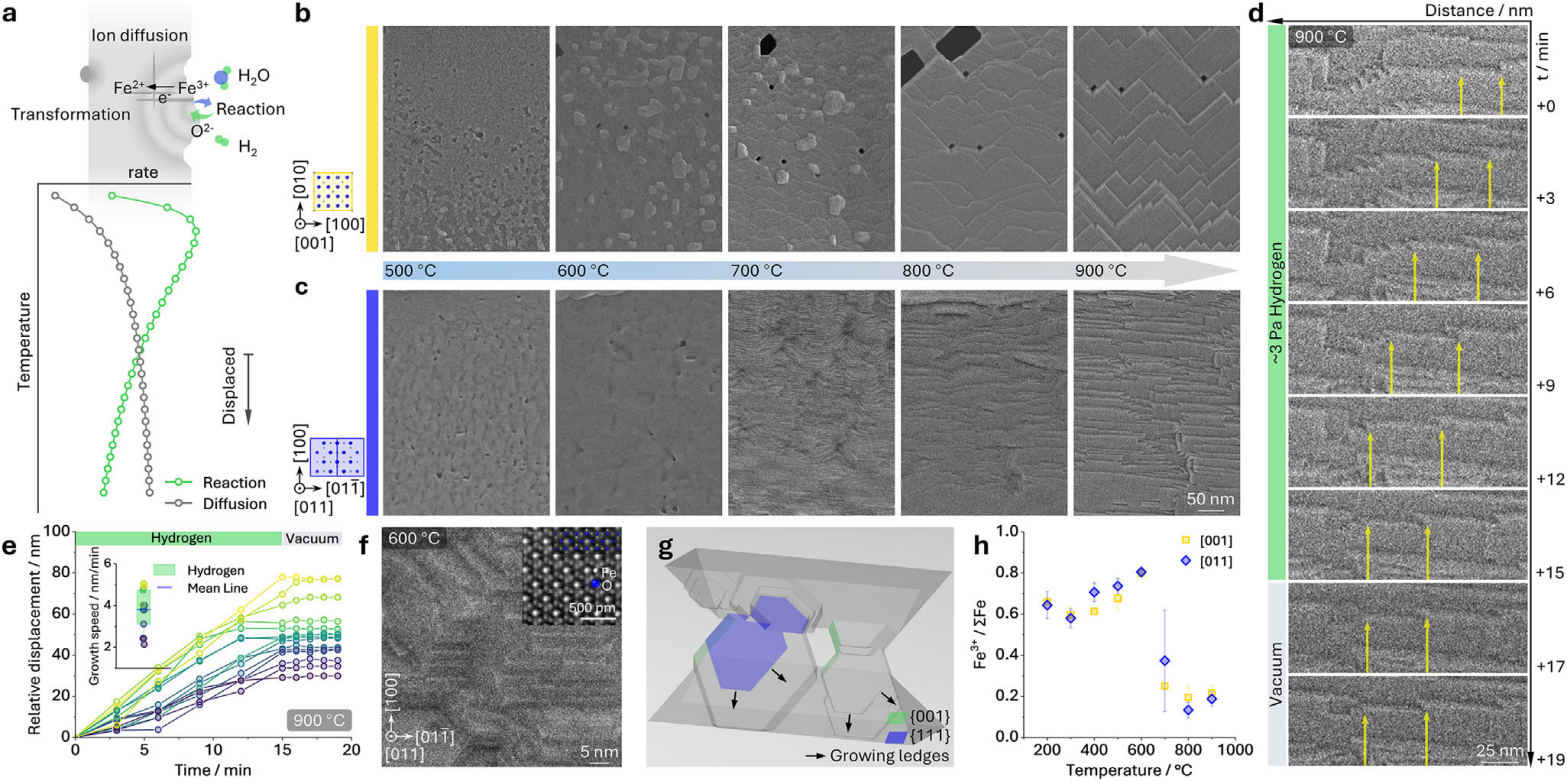
Dynamic surface topological reconstruction via a ledge growth mechanism. (a) A general concept of the displaced reaction‐transformation that occurs when the diffusion rate outperforms the reaction rate in an individual reaction. Here, blue and green spheres represent O and H, respectively, whereas the light‐grey regions denote iron oxides. (b,c) Secondary electron (SE) images showing the surface reconstruction of (b) [001]‐oriented and (c) [011]‐oriented ${\mathrm{Fe}}_{3}O_{4}$ specimens across a temperature range of 500 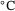 to 900 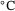 observed in an HF5000 environmental transmission electron microscope (ETEM). Projections of the ${\mathrm{Fe}}_{3}O_{4}$ surface structure and the corresponding crystallographic orientations are shown on the left of each panel. (d) Dynamic surface terrace growth along the ⟨011⟩ direction at 900 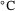 (from top to bottom panel). Two specific ledges indicated by yellow arrows highlight their motion over time in the presence of H_2_, which ceases when the supply of H_2_ is stopped (panels labeled ‘vacuum'). (e) Quantitative analysis of the ledge growth rate, based on the motion of 15 ledges measured at 10 time frames. The inset shows the average measured growth rate. (f) SE image of a [011]‐oriented specimen at 600 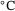, showing abundant surface terraces. The corresponding high‐angle annular dark‐field (HAADF) presented in inset shows the ${\mathrm{Fe}}_{3}O_{4}$ structure. (g) Schematic view of the crystallographic and morphological configurations of [011]‐oriented specimens, illustrating how actively growing {111} planes (in blue) and passively formed {001} planes (in green) are arranged. (h) Ratio of Fe^3+^ (Fe^3+^/ΣFe) extracted from the electron energy loss spectroscopy (EELS) analysis, showing a drop at approximately 700 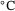, indicating the reduction of the initial ${\mathrm{Fe}}_{3}O_{4}$ to ${\mathrm{Fe}}_{1-x}O$.

Reductions of ionic solids could potentially exhibit displaced reaction–transformation behavior, which strongly depends on the gradient in the chemical potential of anion, cation diffusion and local cation saturation [9, 11]. Displaced reaction–transformations can generate distinct morphologies, such as lath or plate structures in iron oxides [19, 20, 21], or induce growth of characteristic ledges at interfaces [22], for instance during Cu_2_O reduction to Cu [23]. Despite their broad significance, displaced reaction–transformations have received limited attention, primarily due to the requirement for real‐time atomic‐scale characterization to elucidate the spatial decoupling in general in the first place, as well as the underlying atomistic mechanisms, kinetics and topologies.

Here we directly visualize and rationalize displaced reaction–transformation processes during the solid‐state reduction of ${\mathrm{Fe}}_{3}O_{4}$ by H_2_ in a 3 Pa environment over the temperature range of 200 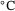
–1000 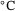, using time‐resolved environmental transmission electron microscopy. By combining bulk reduction results and atomic simulations, we identify ion transport pathways and reveal how morphological evolution coupled with transformation enables displaced reaction–transformation processes. These insights offer a mechanistic framework crucial for precise microstructural and kinetic control in metal‐oxide systems relevant to various redox applications, providing a promising engineering and mechanistic insights for accelerated reduction kinetics beyond macroscopic strategies.

## 2 Results and Discussion

Two representative low‐index oriented lamellae—namely, [001]‐ and [011]‐oriented single‐crystal (SX) ${\mathrm{Fe}}_{3}O_{4}$ were mounted on the same heating chip and annealed according to a specified temperature ramp profile under H_2_ atmosphere (99.999 %, approximately 3 Pa), with temperatures ranging from 200 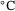 to 1000 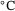 (see Figure S1). Figure 1b presents the surface reconstruction process over the course of the H_2_ reduction of the [001]‐oriented ${\mathrm{Fe}}_{3}O_{4}$ sample starting from 500 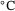. The corresponding high‐angle annular dark‐field (HAADF) images are provided in Figure S2. We also provide the complementary evolution frames in Figure S2a. Upon heating to 700 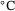, we simultaneously observed distinct ledge and facet structures, along with the presence of surface islands. Here, the evolution of the surface terraces corresponds to the ledge growth process mentioned below. Similarly, we observed this ledge growth process for the [011]‐oriented specimens upon heating up to 700 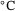 (see Figure 1c). There is no clear evidence for the formation of surface islands when these specimens were heated to temperatures below 700 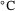 (see the evolution frames up to 400 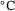 in Figure S2b,c, which has been later also confirmed to be associated with the phase transformation from ${\mathrm{Fe}}_{3}O_{4}$ to ${\mathrm{Fe}}_{1-x}O$ combined with crystallographic surface reconstruction effects. A characteristic feature observed during the reduction of both low‐index surfaces is the emergence of ledge‐controlled growth.

Figure 1d illustrates the dynamic character of the ledge growth process at 900 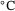, during which the surface terrace advances in the presence of H_2_. Continuous growth of the surface terraces leads to the formation of specifically oriented facets in Figure 1b,c. The ledge growth is driven by the presence of H_2_, rather than by the thermally induced surface reconstruction, as they began to grow when H_2_ was supplied (3 Pa) and immediately ceased growing upon intentionally stopping the H_2_ flow (‘vacuum’ labels on Figure 1d, see Video S1). Figure 1e quantifies the ledge growth kinetics, with an average measured growth rate of approximately 3.8 nm min^−1^ at 900 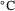. The growth rate varies by approximately 50 % due to height differences among the measured individual terraces.

To quantify the crystallographic orientation relationship of ledge growth within the surface terraces, we combined SE imaging with atomic‐resolution HAADF imaging. The ledges grow on the {111} planes of ${\mathrm{Fe}}_{3}O_{4}$ at 600 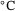 (see experimental image in Figure 1f and schematic illustration in Figure 1g). Diffusion‐mediated atomic rearrangements on {111} planes initiate surface topological reconstructions, during which ledges extend along these planes but are macroscopically confined to the [110] direction (as observed in Video S1) due to the crystallographic orientation and lamellar configuration. Such growth would terminate or advance in the form of a newly formed ledge upon encountering adjacent ledges. This interaction, together with local geometric and interfacial heterogeneity, probably leads to deviations in growth kinetics among individual terraces under a constant volumetric transport flux. At the same time, strain‐modulated Fe‐cation diffusion under these dynamical chemo–mechanical constraints, as well as binding between Fe adatoms and non‐lattice oxygen at the reaction front, may cause nonlinear fluctuations in growth kinetics, as shown in Figure 1e. A similar phenomenon was also observed for the [001]‐oriented specimen when exposed to hydrogen up to 900 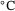 (see Figure S3). This consistent crystallographic feature suggests a thermodynamically driven process, generally applicable to multiple surface orientations. Furthermore, atomic‐resolution annular bright‐field imaging (see Figure S3) reveals a transformation of the crystal structure in the [001]‐oriented specimen to ${\mathrm{Fe}}_{1-x}O$. The electron energy loss spectroscopy (EELS) analysis confirms a phase change occurring at approximately 700 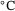, as evidenced by the drop of the Fe^3+^/ΣFe ratio from 0.7 to 0.2 (see Figure 1h) and the disappearance of the characteristic pre‐peaks in the O K‐edge [24] (see Figure S5). The suppression of the O K pre‐peak more plausibly reflects either substructural changes caused by the accumulation of Fe interstitials accompanied by O vacancies at specific sites or the substantial depletion of tetrahedrally coordinated Fe associated with the reduction of ${\mathrm{Fe}}_{3}O_{4}$ to ${\mathrm{Fe}}_{1-x}O$. The crystallographic orientation relationship established for the ledge growth is valid for both ${\mathrm{Fe}}_{3}O_{4}$ and ${\mathrm{Fe}}_{1-x}O$, as well as for reduced ${\mathrm{Fe}}_{3}O_{4}$ ranging from mesoscale synthetic ore down to nanoparticles (see Figure S4, and Experimental Section for details), in which the surface topological reconstruction during H_2_ reduction is realized by such a universal ledge growth mechanism. The topological variance (e.g. size) of ${\mathrm{Fe}}_{3}O_{4}$ perturbs the chemical potential of the species [25], thereby potentially modifying the dynamic thermodynamic response during reduction by altering such as free surface energies. However, the observation of identical reconstruction features across nanoparticles, thin films suggests that the chemical–potential perturbations introduced by size diversity remain within the tolerance of the thermodynamic pathway where the reconstruction still occurs on the {111} plane. We can thus identify a scenario of simultaneously occurring surface ledge growth with phase transformation, suggesting a cascading and coupled mechanism for the reactive transformation, which we elaborate on in the following.

The dynamic H_2_ reduction process reshapes the reaction front and modifies the local boundary conditions that determine surface topology and phase transformation pathways. We show that significant changes in surface morphology of the ${\mathrm{Fe}}_{3}O_{4}$ occur predominantly above 600 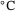. At these temperatures, thermal diffusion is able to drive the crystal toward its equilibrium Wulff shape, generating nanofacets that minimize surface energy [26], for instance forming {111} facets on {110} surfaces of ${\mathrm{Fe}}_{3}O_{4}$ [13]. Thermal diffusion alone, however, cannot explain current results as the motion of ledges almost completely stops when the H_2_ flow is stopped, indicating that surface reactions drive the process. H_2_ indeed removes the surface O, but reconstruction coupled with phase transformation requires redistribution of Fe cations. We hypothesize that the O vacancies result in the accumulation of excess Fe adatoms on the reacting surface. Under the low O partial pressure of our in situ experiments ($P_{O_{2}}\approx 3.4\times 10^{-4}$ Pa), these adatoms preferentially locate on {111} terraces, where they can react with O atoms to produce localized oxidation and propagate the ledge motion. We therefore propose that the hydrogen reduction‐induced coupled migration of Fe cations and residual O drives synchronous ledge growth and phase transformation on the {111} planes. This mechanism requires both mobile Fe atoms and an intact $O^{2-}$ skeleton of the bulk material that serves as a scaffold for newly formed iron oxides, enabling the observed surface reconstruction.

Figure 2a shows the setup of the environmental transmission electron microscopy (ETEM) experiments (see Experimental Section). As schematically shown in Figure 2b, we propose that interstitial (as supported by our defect‐chemistry calculations) Fe cations migrate to the {111} ledges in this type of nanoscale displaced reaction–transformation. These Fe cations primarily originate from {100} planes, positioned at the forefront of the reduction sequence, as the mixed, non‐polar arrangement of Fe and O ions facilitates the removal of O atoms by H_2_. This remains valid even when the {100} surfaces adapt the surface cation vacancy (SCV) reconstruction, as reported in Ref. [27]. Upon O removal from the surface, excess Fe cations diffuse either along the surface or into the bulk, preserving the non‐polar nature of the surface. In contrast, the {111} surface of ${\mathrm{Fe}}_{3}O_{4}$, characterized by alternating Fe and O layers, exhibits polarization. When the {111} surface terminates with octahedrally coordinated Fe atoms (Fe_oct._), it effectively protects the subsurface O atoms from reacting with H_2_ [28]. These additional Fe atoms on the polarized {111} surface can either bond with surface O adatoms or be oxidized by residual O remaining in the column of ETEM to form ${\mathrm{Fe}}_{1-x}O$ layers at the {111} ledges. Figure S3a provides a sketch of the surface geometry along with high‐resolution imaging at 900 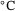, illustrating the crystallographic features of the surface reconstruction. Here, we ascribe the macroscopic mass loss reaction process of SX ${\mathrm{Fe}}_{3}O_{4}$ to a localized yet spatially displaced reaction‐transformation. The surface reaction proceeds only within the outermost few atomic layers, whereas Fe cations can migrate hundreds of nanometers toward interior regions—a spatial displacement of roughly 2–4 orders of magnitude in length scale. Such a process requires the reactant matrix both to exhibit high solubility for interstitial Fe cations and to support their rapid diffusion over long distances in conjunction with a gradient in the chemical potential for oxygen and iron.

**FIGURE 2:**
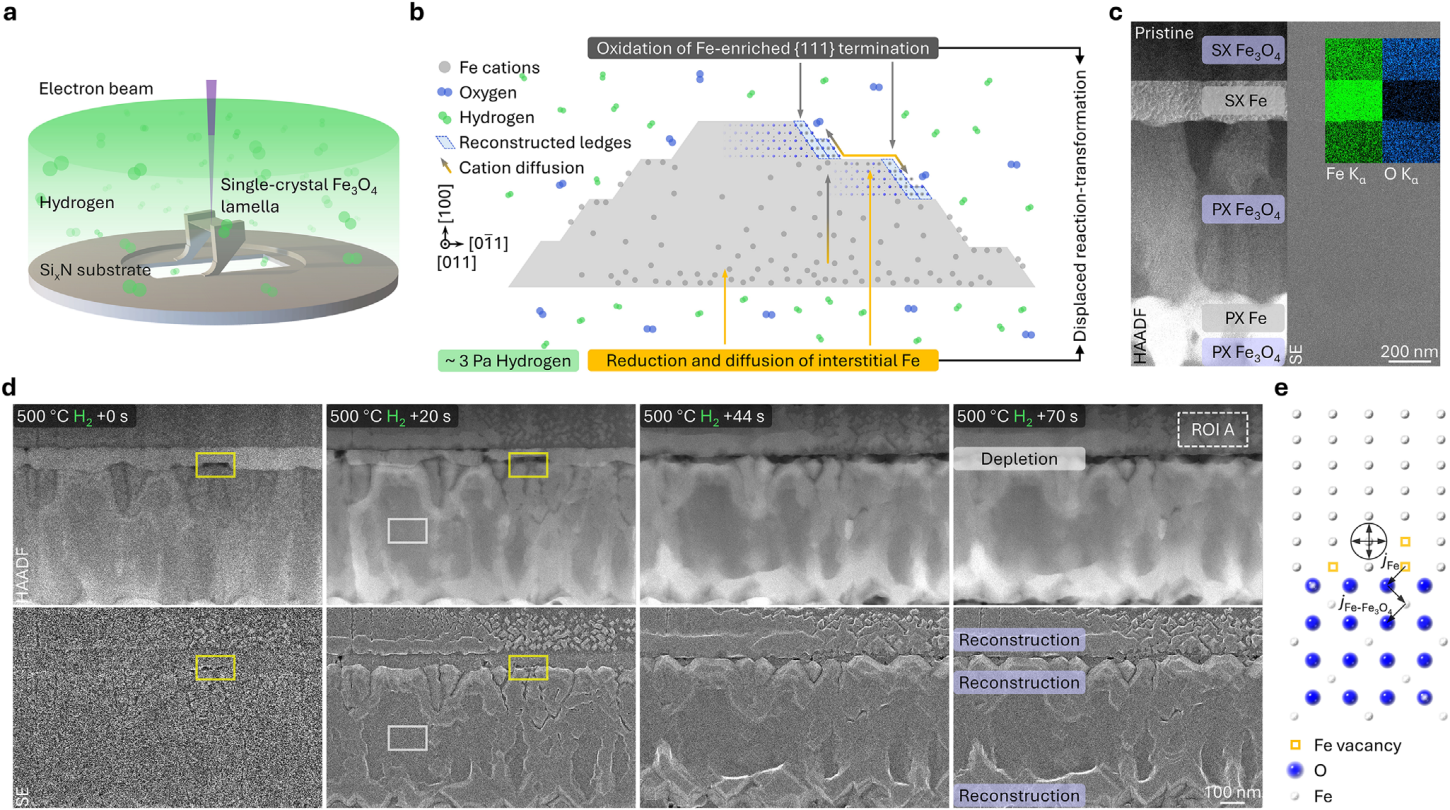
Fast interstitial Fe cation diffusion induces displaced reaction–transformation. (a) Illustration showing the setup of the specimen and the heating chip inside the colum of Hitachi HF5000 ETEM filled with H_2_ (colored in green). (b) Schematic view illustrating the interstitial‐driven displaced reaction‐transformation in hydrogen‐based direct reduction (HyDR) of ${\mathrm{Fe}}_{3}O_{4}$. Displaced reaction–transformation during reduction (through ledge growth) occurs separately on different surfaces. The orange arrow indicates the diffusion of interstitial Fe toward the Fe‐enriched O‐rich {111} surfaces, where oxidation subsequently forms surface ${\mathrm{Fe}}_{1-x}O$ layers, as indicated by the gray arrows. Fe cations are represented in gray, O atoms in blue, and H atoms in green. Reconstructed terraces are indicated by hatched areas. (c) HAADF and SE images of the pristine multilayered Fe/Fe_3_O_4_ lamella. Here, SX and PX refer to single‐crystal and polycrystal regions, respectively. Overlaid on the SE image are energy‐dispersive X‐ray spectroscopy (EDS) compositional maps of Fe $K_{\alpha }$ and O $K_{\alpha }$. (d) In situ ESTEM hydrogen‐based reduction of the multilayered lamella at 500 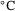. Nanovoids form within 20 s, and the depletion of metallic Fe layer within 44 s, along with enhanced reconstruction and terrace formation on the adjacent Fe_3_O_4_ layer. The yellow and white rectangles indicate representative regions at the Fe/Fe_3_O_4_ interface and within the Fe_3_O_4_ layer, respectively. (e) Atomic model illustrating the mechanism for nanovoid formation at the Fe/Fe_3_O_4_ interface. When the Fe self‐diffusion flux in bulk BCC Fe ($j_{\mathrm{Fe}}$, indicated by the black circle) fails to offset the interfacial Fe flux ($j_{\mathrm{int}}$), vacancies coalesce into voids as metallic Fe atoms transform into interstitial cations in the oxide. Fe atoms are depicted in gray, O atoms in blue, and Fe vacancies are indicated by yellow squares.

In this sense, and to probe the transport behavior of Fe, we intentionally build up an O chemical potential gradient to induce directional cation migration. Time‐resolved frames depict the evolution of the multilayered Fe/Fe_3_O_4_ lamella (Figure 2c) under an H_2_ pressure of approximately 3 Pa at 500 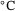 (Figure 2d). Nanovoids emerge immediately at the Fe/Fe_3_O_4_ interface upon H_2_ injection. Focusing on the species and charge transfer at the phase interface schematically shown in Figure 2e, the Kirkendall voids [29] generated by vacancy aggregation confirm the defect transport across the interface (detailed in Experimental Section). After 70 s, the Fe layer is depleted (see Figure S6), and substantial surface reconstruction emerges in Fe_3_O_4_ regions adjacent to the Fe layer, which is much more pronounced than in areas further away from the interface (see also Video S2). Fe depletion proceeds through an initial burst that lasts up to 44 s, during which pronounced reconstruction develops on the surface of Fe_3_O_4_. It is challenging to track down the origin of interfacial void formation, since this process is accompanied by structural modifications and dynamically changing chemical conditions. However, the depletion of the Fe layer, reconstruction continues at a lower rate and gradually propagates away from the initial Fe layer as a wave‐like front (see Video S3). In contrast, in the absence of an additional Fe layer, surface reconstruction proceeds in a non‐directional and isotropic manner, as shown in Figure 1b,c and Figure S7 and Video S4. This directional wave‐like surface reconstruction strongly suggests a possible directional migration of excess Fe cation that most possibly diffused from Fe layer (supported by the depleted Fe layer), demonstrating the high penetrability and fast diffusion of Fe cations in Fe_3_O_4_, supporting the Fe cation–dominated reaction‐transformation mechanism. Local enrichment of Fe cation interstitials coordinates the displaced reaction–transformation, resulting in a counterintuitive surface ledge growth on {111} planes, and phase transformation in the low‐pressure H_2_ environment.

To further investigate and validate the proposed underlying mechanism, we performed atomistic simulations using our recently developed atomic cluster expansion (ACE) interatomic potential for the Fe‐O system [30]. Under conditions where the products can be continuously removed by the constant hydrogen flow with stable gas diffusion, we briefly map the experimental atmospheric pressure to the corresponding oxygen chemical potential within the equilibrium thermodynamic framework and discuss the stability of defect phases and surfaces. As observed during the in situ reduction, and sketched in Figure 3a,b, the ledges move through the formation of ${\mathrm{Fe}}_{1-x}O${111} layers on Fe_3_O_4_{111} surfaces exposed to the weakly reducing environment. The orientation of growing ${\mathrm{Fe}}_{1-x}O$ layers is first dictated by the energy of the exposed surface termination. In this respect, we present in Figure 3c the surface phase diagram of both ${\mathrm{Fe}}_{1-x}O$ and Fe_3_O_4_ considering the most relevant terminations of both {001} and {111} surfaces. In all presented simulations, we use the stoichiometric form of wüstite, FeO. Across the experimentally accessible range of O chemical potentials, the FeO{111}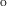 is the most stable surface, with an approximately 0.02‐0.1 eV Å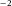 lower energy than the O‐rich facet of Fe_3_O_4_{111}. One would thus expect FeO{111} facets to grow instead of additional {111} layers of Fe_3_O_4_. On the other hand, these growing FeO layers will create interfaces with bulk Fe_3_O_4_ (see Figure S8 and Experimental Section). The relative stability of the different interfaces as a function of the O chemical potential $\Delta \mu_{O}$ is presented in Figure 3d. We also present in Table S1, the O chemical potential stability range of each configuration of the FeO/Fe_3_O_4_ interface, as well as their adhesion energy $\gamma_{\text{adh.}}$. The {111} interface is energetically most favorable across the whole range of the O chemical potential, as compared to the {100} interface, supporting the preferential growth of FeO on {111} surfaces of Fe_3_O_4_.

**FIGURE 3:**
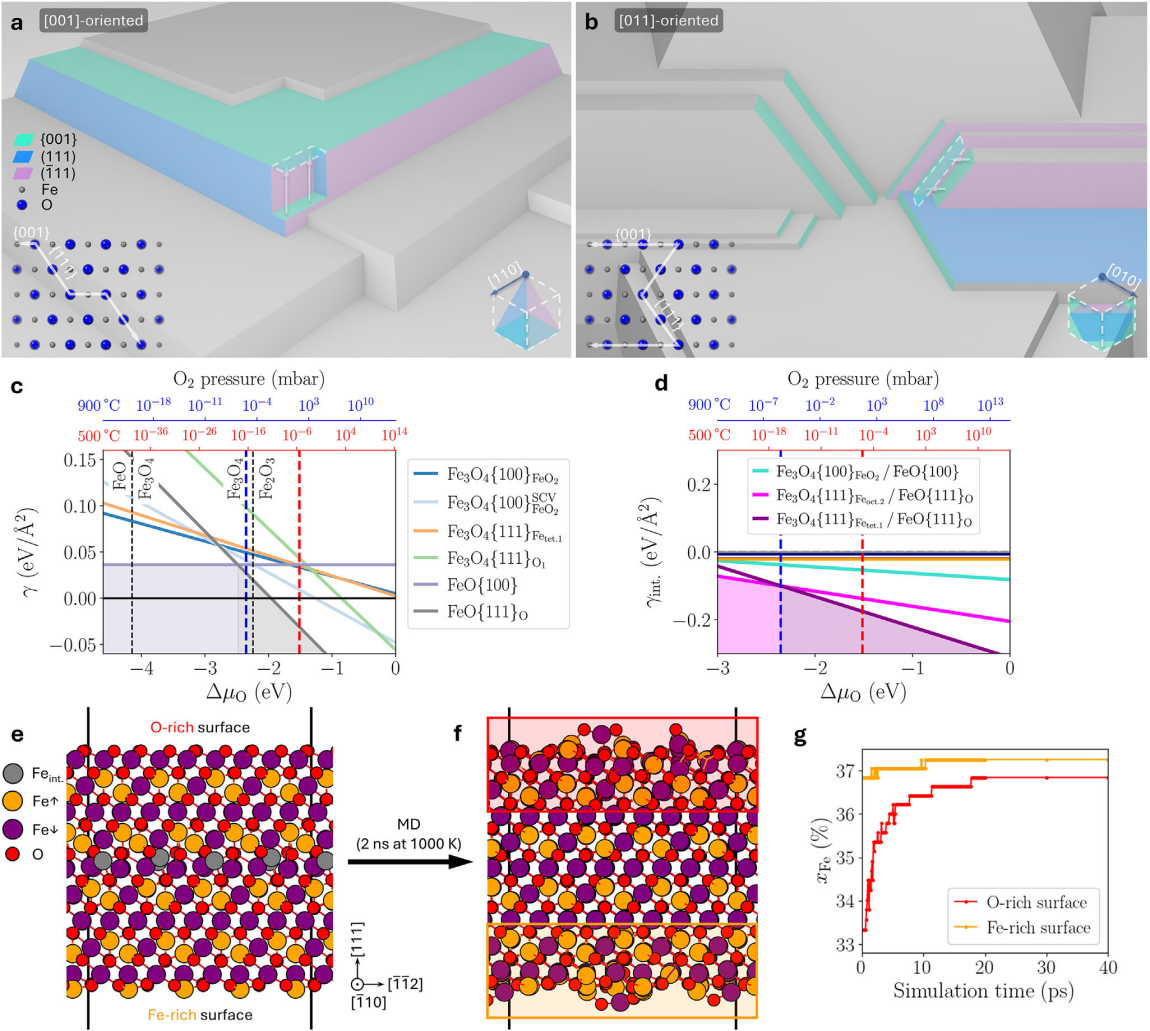
Ledge growth mechanism and interstitial Fe cation diffusion. (a,b) Sketch of the ledge growth mechanism of surface terraces observed during in situ HyDR experiments on (a) the [001]‐oriented and (b) the [011]‐oriented Fe_3_O_4_ samples (see Figure 2b). Surface orientations are color‐coded, with {001}, {111}, and {111} surfaces indicated by green, blue, and purple, respectively; Fe atoms are represented in gray and O atoms in blue. (c) Surface phase diagram of FeO and Fe_3_O_4_ as a function of the O chemical potential change $\Delta \mu_{O}$ (referenced to molecular O_2_). The black vertical dashed lines indicate the stability ranges of the different iron oxides, while vertical red and blue lines indicate the O chemical potentials estimated from the experimentally derived partial pressure of oxygen in the ETEM ($3.4\times 10^{-4}$ Pa) at 500 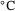 and 900 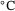, respectively. (d) FeO/Fe_3_O_4_ interface phase diagram as a function of $\Delta \mu_{O}$ (interface structures are presented in Figure S8). (e,f) Snapshots of a molecular dynamics (MD) simulation at 1000 K (727 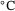) of interstitial Fe atoms diffusing to Fe_3_O_4_{111} surfaces, at (e) t=0 ns and (f) t=2 ns, respectively. In the Fe_3_O_4_ structure, Fe atoms are color‐coded by spin orientation, with spin‐up atoms colored orange and spin‐down atoms purple. Interstitial Fe atoms are depicted in gray, and oxygen atoms are shown in red. (g) Evolution of the surface Fe concentration $x_{\text{Fe}}$ as a function of simulation time in the two O‐rich (red) and Fe‐rich (orange) surface regions highlighted by shaded color boxes in (f). Compared to the Fe‐rich surface, the Fe interstitial concentration on the O‐rich side increases from 33.4 at.% to 36.8 at.%, confirming the tendency of interstitial Fe cations to diffuse to regions of high O chemical potential.

In the reducing H_2_ environment, the concentration of Fe interstitial cations tends to increase inside the bulk Fe_3_O_4_ before the nucleation of metallic Fe, proceeding along the removal of O atoms by H_2_. This process can be expressed as:

(1)

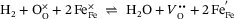




To probe the diffusion of interstitial Fe cations (${\mathrm{Fe}}_{\text{int.}}$) to the {111} surface of Fe_3_O_4_, we implanted ${\mathrm{Fe}}_{\text{int.}}$ cations at the middle of a slab model exposing both the most stable Fe‐rich and O‐rich terminations of the {111} surface of Fe_3_O_4_ on each of the two sides of the slab (Figure 3e). In the first tens of picoseconds of the moelcular dynamics (MD) simulation, ${\mathrm{Fe}}_{\text{int.}}$ cations diffuse toward the open surfaces present in the simulation cell. The final configuration of the system after the simulated annealing is presented in Figure 3f. This diffusive motion of ${\mathrm{Fe}}_{\text{int.}}$ cations occurs predominantly toward the initially O‐rich surface of the slab, resulting in an Fe enrichment in this region of the cell. After annealing, the originally O‐rich surface now shows a higher Fe concentration (Figure 3g). From the computed effective diffusivity of ${\mathrm{Fe}}_{\text{int.}}$ directly from MD simulations, we estimate a characteristic diffusion length of approximately 83 μm within 1 s at 1000 K, thus allowing interstitial Fe cations to quickly diffuse from the O‐poor region of the sample to the O‐rich region (detailed in Experimental Section). The formation energy of Fe adatoms on different surfaces and terminations of both Fe_3_O_4_ and FeO (see Table S2 and Figure S9, further support that Fe adatoms preferentially sit on O‐rich Fe_3_O_4_{111} surfaces under similar chemical potentials. These Fe atoms located on {111} facets of Fe_3_O_4_ can further bond with surface adatoms of O or get oxidized due to residual O contained in the ETEM, gradually forming FeO {111} surface layers on top.

Using atomistic simulations, we thus support our experimental observation that Fe cations initiate the displaced reaction‐transformation during HyDR of Fe_3_O_4_ via directed long‐range diffusive motion. Nanoscale SX Fe_3_O_4_ lamellae constitute a constrained medium, facilitating rapid redistribution of Fe cations before most available vacancies become occupied. However, a larger specific surface area likely reduces the average diffusion path of Fe cations, causing them to preferentially remain at the surface instead of overcoming higher energy barriers to diffuse into the bulk. Next, we further demonstrate the long‐range diffusion of Fe cations and the displaced reaction–transformation within microscale iron oxide ore particles, offering much less surface area and thus allowing us to probe its impact on the bulk reduction kinetics.

Figure 4a,b shows the microscale Fe_3_O_4_ ore reduced at 900 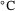 in a $1\times 10^{5}$ Pa H_2_ environment. The orientation and phase mapping obtained through electron backscatter diffraction (EBSD) reveal a distribution of plate‐shaped ${\mathrm{Fe}}_{1-x}O$ inclusions in the Fe_3_O_4_ matrix bearing a cube‐on‐cube relationship with the host [31], spreading three‐dimensionally and penetrating tens of micrometers into Fe_3_O_4_ (supported by Figure S10a). The atom probe tomography (APT) analysis presented in Figure 4c reveals the variations in composition measured across an interface between Fe_3_O_4_ and ${\mathrm{Fe}}_{1-x}O$, where the phase boundary is visualized with a 10 at.% isosurface of O_2_^+^ species, and a transition in Fe and O contents is evident across the interface. Treating the surface O chemical potential as that of metallic Fe—an upper limit—and the interior as that of bulk Fe_3_O_4_ yields a maximum 0.9 eV difference across 5‐20 μm at 900 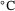 and $1\times 10^{5}$ Pa H_2_, corresponding to a gradient $\partial \mu_{O}/\partial x$ of 0.5–1.8$\times 10^{5}$ eV $m^{-1}$. Such reactive phase transformation takes place tens of micrometers away from the reaction surface where O atoms are removed from the lattice by H_2_, aligning well with the in situ nanoscale displaced reaction‐transformation. Notably, under high‐pressure hydrogen reduction of bulk Fe_3_O_4_, the faster reduction kinetics build up an intensified O chemical potential gradient with Fe cation enrichment, which is not sufficient to increase the diffusion resistance, driving large‐scale Fe cation migration and the phase transformation front deep into the bulk instead.

**FIGURE 4:**
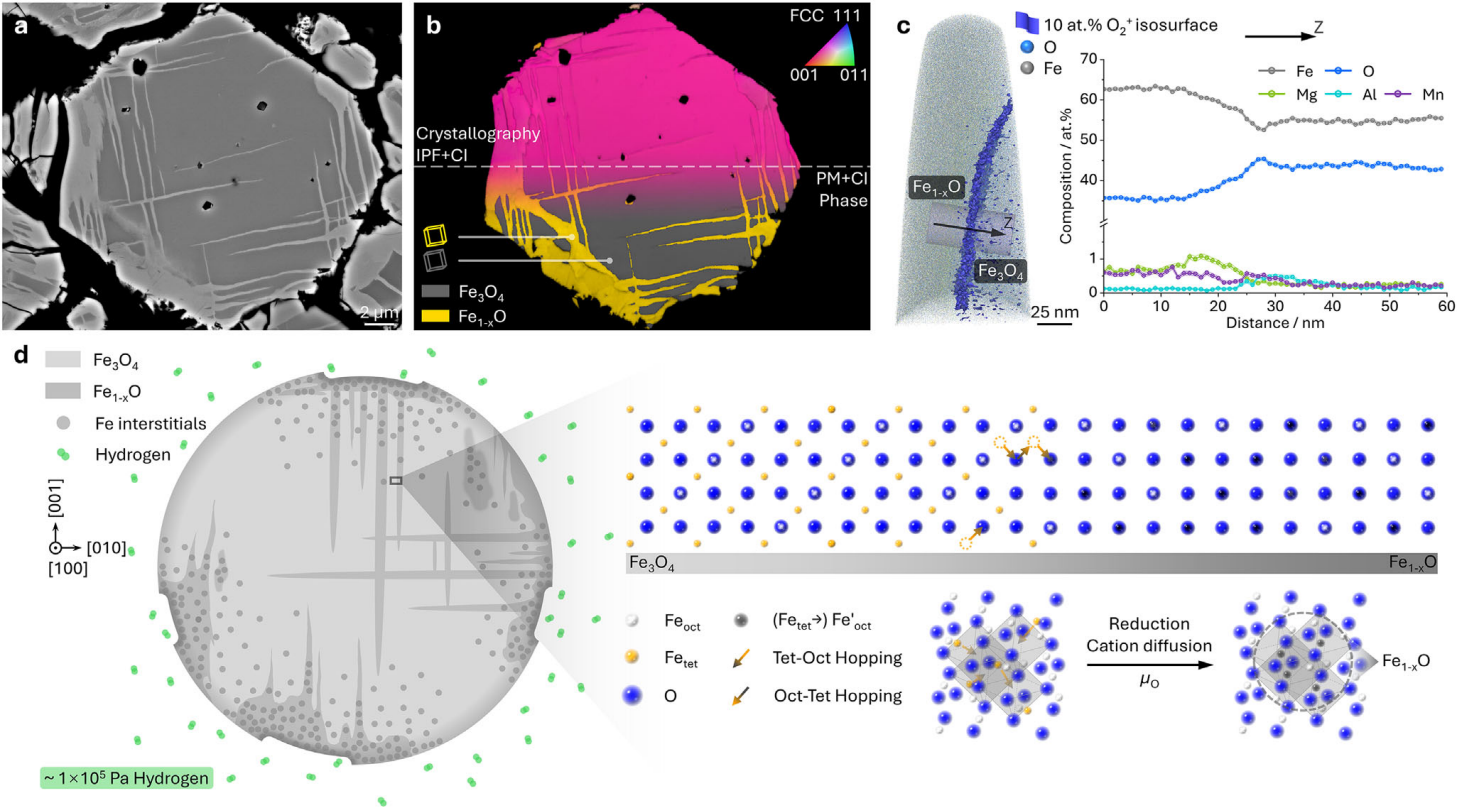
A three‐dimensional net‐plate‐like structure forms during the H_2_ reduction of bulk Fe_3_O_4_ ore. (a,b) (a) Backscattered electron (BSE) and (b) electron backscatter diffraction (EBSD) images showing the three‐dimensional net‐plate‐like distribution of ${\mathrm{Fe}}_{1-x}O$ in the Fe_3_O_4_ matrix. Here, the EBSD map combines a phase map (bottom) and an orientation map (top), revealing the ${\left(001\right)}_{{\mathrm{Fe}}_{3}O_{4}}$//${\left(001\right)}_{{\mathrm{Fe}}_{1-x}O}$, ${\left[001\right]}_{{\mathrm{Fe}}_{3}O_{4}}$//${\left[001\right]}_{{\mathrm{Fe}}_{1-x}O}$ crystallographic orientation relationship (also see Figure S10a). (c) Atom probe tomography (APT) analysis of the phase boundary between Fe_3_O_4_ and ${\mathrm{Fe}}_{1-x}O$. The phase boundary is highlighted by the 10 at.% iso‐surface of O_2_^+^ ions. Elemental partitioning of gangue elements (Al, Mg) occurs across the boundary, with compositions quantified using a one‐dimensional profile. (d) Sketch of the formation mechanism of three‐dimensional net‐plate‐like structured ${\mathrm{Fe}}_{1-x}O$ inside the Fe_3_O_4_ matrix. The removal of the O lattice atoms by environmental H_2_ at the surface results in Fe atom enrichment, driving the interstitial diffusion of Fe cations into the bulk and the subsequent localized formation of ${\mathrm{Fe}}_{1-x}O$. The enlarged sketch (right, top) illustrates interstitial diffusion and localized ${\mathrm{Fe}}_{1-x}O$ formation. The illustration (right, bottom) highlights the energetically favorable hopping of Fe cations from tetrahedral sites (colored grey) to octahedral sites labeled with prime symbols (colored gold). O and H atoms are colored blue and green, respectively.

We sketch in Figure 4d the microscale reduction mechanism from Fe_3_O_4_ to ${\mathrm{Fe}}_{1-x}O$ at atomic scale, illustrating typical noncollinear yet percolating hopping of atoms and the associated local phase transformation [32, 33]. The large interior volume of the Fe_3_O_4_ oxide, positioned away from the reduction surface, provides sufficient space for the relocation of Fe cations. Fe cations tend to migrate into the bulk oxide rather than remain on the surface, due to the relatively higher O chemical potential in the former, as confirmed by our simulations (Figure 3e,f). Migration of Fe atoms locally alters the fugacity of cations in the O lattice, finally adapting to the thermodynamically preferred phase. A likely outcome is that the redistribution of Fe in a localized non‐equilibrium state results in the formation of chemical medium‐range and even short‐range ordered substructures (such as Koch–Cohen defect clusters [34]). With a local increase in concentration of interstitial Fe cations in Fe_3_O_4_, filling the majority of the energetically favorable octahedral sites through Fe ions jumping from adjacent tetrahedral sites (as both follow the crystal field stabilization energy rules), ${\mathrm{Fe}}_{1-x}O$ can locally form. Such transformation conserves the FCC $O^{2-}$ lattice, while the Fe cations redistribute in the form of Frenkel cluster migration, resulting in a diffusion‐mediated migration of the inter‐phase boundary that is spatially displaced from the reduction surface. Cation‐induced phase transformation has been previously reported during the redox reaction between ${\mathrm{Fe}}_{2}O_{3}$, Fe_3_O_4_ and ${\mathrm{Fe}}_{1-x}O$ [19, 20, 21], representing a general phenomenon that can occur in various systems and at different stages of the reduction process.

## 3 Conclusion

In summary, the Fe cation‐mediated displaced reaction–transformation is a general and fundamental mechanism occurring during HyDR of iron oxides, such as Fe_3_O_4_, as confirmed by both in situ and ex situ bulk analyses. It requires a high propensity for cation diffusion, which in turn requires the presence of a vacancy‐rich structure capable of rapidly transporting and accommodating cations while maintaining lattice stability. We anticipate that such a displaced reaction–transformation mechanism dominated by cation transport would be broadly applicable to other thermodynamically reducible spinel‐structured oxides (with a prototypical AB_2_O_4_ formula), especially for the Fe‐group cations (with A and B belonging to, e.g., Fe, Co, and Ni) due to their similar diffusion behavior and valence characteristics. Incorporation of this mechanism offers engineering strategies to tune reduction kinetics—for example, by constructing cation transport shortcuts (grain boundaries, dislocations, or alloying elements) or designing targeted microstructures, such as faceted surfaces, to facilitate surface catalytic reactions. The reduction of iron oxides is a multi‐step process. In the case of reducing ${\mathrm{Fe}}_{1-x}O$ (or other halite‐structured oxides), where the material is already saturated in Fe interstitial cations compared to Fe_3_O_4_, the role of cations in governing reduction kinetics progressively diminishes. As structural changes accumulate during reduction, one must carefully design and optimize each reduction step individually to optimize the overall reaction kinetics of the process. They do not only shed light on the atomistic mechanisms behind efforts to make a 2 billion annual ton industry more sustainable, but also give hints on potentially similar effects occurring in other solid state redox systems, such as corrosion, dealloying and batteries.

## 4 Experimental Section

### 4.1 Material Synthesis and Fabrication

Fe_3_O_4_ is an intermediate oxide phase formed during the solid‐state hydrogen‐based reduction of ${\mathrm{Fe}}_{2}O_{3}$. To isolate cation‐mediated transformations while eliminating reaction‐history effects and avoiding additional crystallographic effects or volume changes, we break the problem down here into the Fe_3_O_4_ to ${\mathrm{Fe}}_{1-x}O$ reduction step, which exhibits relatively small volume contraction (approximately 20 vol.%) while preserving the underlying structure of the oxygen sublattice. We synthesized SX Fe_3_O_4_ thin films on the (001) plane of SX MgO substrates via epitaxial growth using a reactive magnetron sputtering technique [35]. Performing at 300 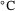 with direct current mode (150 W), a commercial Fe target (Sindhauser, 99.99 wt%) was used to reactively sputter the SX Fe_3_O_4_ films in Ar and O_2_ flow (20:1, 0.5 Pa). For the multilayered thin film, we periodically injected and retracted the O with identical pressure and flow rate at 300 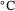, to enable the Fe and Fe_3_O_4_ to epitaxially grow on the MgO substrate. The formation of polycrystalline layer is caused by accumulation of defects and increased heterogeneity at Fe/Fe_3_O_4_ interfaces. Fe_3_O_4_ synthetic ore was synthesized via spark plasma sintering (FCT HP D 5) at 900 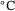 for 30 min. The synthesized samples were ground after sintering so that all ${\mathrm{Fe}}_{1-x}O$ that formed on the outer surface (due to reaction with graphitic elements in the mold) were removed resulting in pure Fe_3_O_4_, then manually pulverized into micropowders. Fe_3_O_4_ ore powders were partially reduced into ${\mathrm{Fe}}_{1-x}O$ by heating to 900 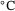 at a rate of 5 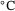 $s^{-1}$ and then immediately cooled down with an infrared furnace in a hydrogen (99.999 %) environment at $1\times 10^{5}$ Pa. Nanoparticles used for in situ direct reduction, approximately 50–100 nm in size, were supplied by Sigma–Aldrich. Lift‐out of specimens were carried out using focused ion beam (FIB) system equipped with Xe plasma source (FEI Helios PFIB). MgO substrate attached to the cross‐section specimen was meticulously cleaned out. Specimens were mounted on semi‐conductive heating chips which are in an overall 5×5 mm cubic shape (NORCADA G3.5). Si_x_N substrate on the heating chip was additionally milled to create rectangle electron‐transparent windows that meet the dimensions of ESTEM specimens (Figure S1c). W deposition source was used throughout the FIB fabrication process to avoid possible catalytic effect rising from Pt or other deposition source. Specimen thinning process was accomplished after all the mounting process, directly on the chip at 30 kV on FIB equipped with Ga source (FEI Helios Nanolab 600i) by enabling a parallel geometry between specimen and plasma gun. Compensate angles varied from 1.5 to 2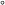 were used to calibrate the milling slope throughout the thinning process in which specimens were finally thinned to less than 80 nm. Due to the operational discrepancies, the final thickness of thinned specimens differed from each other but ended up within the range of 40–70 nm. To decrease the specimen surface damage and contamination, the cleaning process was applied using a Ga source FIB operated at 5 kV and 7.7 pA for 2–3 min per side. Specimens for atom probe tomography were prepared using site‐specific lift‐out procedure, followed by FIB annular milling till the crown radius was less than 100 nm. A final cleaning process of the tips was performed at 5 kV and 16 pA.

### 4.2 In Situ Reduction and Characterization

Specimens were cleaned under ultraviolet (UV) light at 10^4^ Pa for 10 min in a Hitachi ZONETEM II sample cleaner before being inserted into the ETEM. In situ H_2_ reduction experiments were performed on a Hitachi HF5000 microscope equipped with a probe corrector operating at 200 kV with E‐STEM mode. Ultrapure H_2_ (99.999 %) was injected into the column at a constant mass flow of 3 ${\mathrm{cm}}_{STP}^{3}$ $\min^{-1}$, responsible for the column pressure of around $2.7\times 10^{-2}$ Pa and specimen pressure of around 3 Pa (according to the calibration of the instrumental intrinsic configurations). Embedded spiral heating wires and calibrated parameter settings allow a homogeneous and stable thermal condition, as the preset and real‐time temperature captured by monitor software (Blaze) showed negligible temperature deviation (see Figure S1a). Importantly, chips were pre‐heated at 200 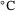 inside the column of the microscope for 15 min to prevent specimens from possible carbon redeposition and contamination in all groups of in situ reduction experiments. Electron energy loss spectrum (EELS) were recorded at STEM‐EELS mode using CEOSS CEFID system, zero‐loss and element‐specific edges (Filter window ranged from 494–750 eV to include both Fe and O edges) were acquired separately. Quantification analysis of Fe^3+^ concentration (Fe^3+^/ΣFe) was performed on the L_3,2_ energy‐loss near‐edge structures using integral intensity evaluation method based on the calculation of L_3_/L_2_ ratio [36]. EELS were acquired at three random points for each temperature plateau. Backscattered electron (BSE) imaging and electron backscatter diffraction (EBSD) were performed on Carl Zeiss Merlin scanning electron microscope operating at 10 kV and 2.2 nA. Scan data were analyzed using OIM Analysis 9 by performing spherical re‐indexing process, which expresses the experimental Kikuchi bands in spherical harmonics [37]. A series of processes involving spherical harmonics cross‐correlation searches for the best match is then performed to minimize the deviation between the experimental and simulated Kikuchi bands. A confidence index threshold of 0.5 was selected to properly visualize the orientation and phase mapping in Figure 4b (see Figure S10c). Evaporation events for atom probe tomography analysis were collected with a reflectron‐fitted instrument (LEAP 5000XR) at 50 K base temperature, with a near‐ultraviolet (λ = 355 nm) laser illuminating at 40 pJ and 200 kHz, and a detection rate of 0.006 detected ions per pulse. Data reconstruction and analysis were performed using IVAS software integrated in AP Suit 6.3. It should be noted that a significant O deficiency occurs during oxide evaporation due to the molecular dissociation of ionized O‐contained parent fragment and formation of O_2_ neutrals [38]. Corresponding mass spectrum ranging from 0 to 110 (Mass charge ratio, Da) can be found in Figure S10d.

### 4.3 Thermodynamics and Kinetics of Cross‐Interface Transport

Figure 2e presents the schematic atomic transport process across the interface with the ${\left(100\right)}_{{\mathrm{Fe}}_{3}O_{4}}$//(100)_Fe_, ${\left[001\right]}_{{\mathrm{Fe}}_{3}O_{4}}$//[110]_Fe_ orientation relationship. The depletion of the pure Fe layer observed under the H_2_ environmental conditions suggests the presence of a net Fe cation flux aligned with the interfacial gradient, consistent with chemically driven directional interdiffusion, where the gradient of the O chemical potential at the iron–oxide interface supplies a thermodynamic driving force that promotes Fe migration from the metallic layer into the oxide. Here, *j*_Fe_ and $j_{\mathrm{Fe}-{\mathrm{Fe}}_{3}O_{4}}$ denote the spatially homogeneous self‐diffusion flux of Fe in bulk metallic Fe and the Fe flux in bulk Fe_3_O_4_, respectively (see Figure 2e). The diffusion coefficient of Fe in Fe_3_O_4_ lies between 10^−20^ to 10^−19^ m^2^ s^−1^ at 500 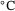 [39, 40], depending on the O partial pressure, which exceeds the self‐diffusion rate in body‐centered‐cubic (BCC) Fe ($7.738\times 10^{-23}$ m^2^ s^−1^ at 500 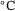, according to the Database MOBFE6 of Thermo‐Calc2024a). It turns out that *j*_Fe_ is 3‐4 orders of magnitude lower than $j_{\mathrm{Fe}-{\mathrm{Fe}}_{3}O_{4}}$ as the flux is proportional to the diffusion coefficient. The resulting unbalanced net flux of Fe leads to the formation of Kirkendall voids, as observed in Figure 2d. The cation diffusivity diagram of Fe_3_O_4_ [41] shows that Fe vacancies govern Fe cation diffusion under O‐rich (oxidizing) conditions, whereas Fe interstitials dominate under O‐poor (reducing) conditions. The present H_2_ environment therefore promotes Fe‐interstitial diffusion.

The overall process can be written using the Kröger–Vink notation as follows:

(2)

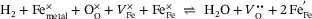

Additional sub‐processes can also occur through the Frenkel defect reaction describing the hopping between tetrahedral and octahedral sites with the transfer of an electron:

(3)

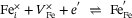

For those reactions, the electron donor can either be metallic Fe or H_2_.

### 4.4 Atomistic Simulations

All atomistic simulations performed in this work are carried out using the large‐scale atomic/molecular massively parallel simulator (LAMMPS) code [42] and an ACE [43, 44] interatomic potential developed for the entirety of the Fe‐O binary system [30]. We demonstrated the ability of the model to accurately reproduce bulk and defect properties (e.g. vacancies, interstitials, surfaces…) of both pure Fe and its oxides.

Per construction, this ACE potential for Fe‐O has an explicit account of magnetic degrees of freedom of the Fe sub‐lattice, through the definition of three different species for Fe atoms depending on the sign of their magnetic moments (see details in Ref. [30]). This allows one to equilibrate the magnetic configuration of the system on‐the‐fly while performing classical molecular dynamics (MD) simulations. Equilibration of the Fe spins is done via semi Grand Canonical Monte–Carlo swaps between different “species” of Fe atoms at each timestep of the MD run. In the present work, we used this type of coupled dynamic simulations to study diffusion of interstitial Fe atoms, both in bulk Fe_3_O_4_ and to its surfaces (Figure 3e–g).

#### 4.4.1 Surface Phase Diagrams

Surface phase diagrams (e.g. Figure 3c) are constructed considering all possible bulk‐truncated surface terminations of the investigated surface orientations (here, the {100} and {111} facets of Fe_3_O_4_ and FeO). Out of all these terminations, only the most relevant (i.e. with the lowest energy in the O‐rich region) are plotted on Figure 3c.

Surface energies $\gamma_{\text{surf.}}$ of different facets are computed as a function of the O chemical potential change $\mu_{O}=\mu_{O}^{O_{2}}+\Delta \mu_{O}$, with $\mu_{O}^{O_{2}}$ the energy of an O atom in an O_2_ molecule. For surfaces of Fe_3_O_4_, we use the following definition:

(4) $$\begin{array}{c} \gamma_{\text{surf.}}^{{\text{Fe}}_{3}O_{4}}\left(\mu_{O}\right)=\frac{E_{\text{surf.}}-\frac{1}{3}\;n_{\text{Fe}}^{{\text{Fe}}_{3}O_{4}}\;E_{{\text{Fe}}_{3}O_{4}}+\left(\frac{4}{3}\;n_{\text{Fe}}^{{\text{Fe}}_{3}O_{4}}-n_{O}^{{\text{Fe}}_{3}O_{4}}\right)\;\mu_{O}}{2\;S}, \end{array}$$

where $E_{\text{surf.}}$ is the total energy of the symmetric slab containing two identical surfaces of area S, $E_{{\text{Fe}}_{3}O_{4}}$ is the energy of bulk Fe_3_O_4_ per formula unit, and $n_{\text{Fe}}^{{\text{Fe}}_{3}O_{4}}$ and $n_{O}^{{\text{Fe}}_{3}O_{4}}$ are the number of Fe and O atoms contained in the slab, respectively. Surface energies for FeO were computed using the following definition:

(5) $$\gamma_{\text{surf.}}^{\text{FeO}}\left(\mu_{O}\right)=\frac{E_{\text{surf.}}-n_{\text{Fe}}^{\text{FeO}}\;E_{\text{FeO}}+\left(n_{\text{Fe}}^{\text{FeO}}-n_{O}^{\text{FeO}}\right)\;\mu_{O}}{2\;S},$$

where $E_{\text{FeO}}$ is the energy of bulk FeO per formula unit, and $n_{\text{Fe}}^{\text{FeO}}$ and $n_{O}^{\text{FeO}}$ are the number of Fe and O atoms contained in the slab, respectively.

On the surface phase diagram of Figure 3c, as well as on the interface phase diagram of Figure 3d, we also converted the oxygen chemical potential $\mu_{O}$ to oxygen partial pressure $P_{O_{2}}$. To do so, we use the following relation:

(6) $$\begin{array}{c} \mu_{O}\left(P_{O_{2}},T\right)=\frac{1}{2}\left[E_{O_{2}}+\mu_{O}^{\text{chem.}}\left(T\right)+k_{B}T\;\ln\left(\frac{P_{O_{2}}}{P^{0}}\right)+E_{O_{2}}^{\text{overbinding}}\right], \end{array}$$

with $E_{O_{2}}$ the energy of an isolated O_2_ molecule as predicted by the ACE potential, $\mu_{O}^{\text{chem.}}$ is the chemical potential of oxygen in gas phase as a function of temperature, taken from thermodynamic tables [45], and $P^{0}$ is the standard pressure (here, $P^{0}=1.013\times 10^{5}$ Pa). $E_{O_{2}}^{\text{overbinding}}$ is a correction to account for the overbinding of the O_2_ molecule predicted by DFT within the GGA‐PBE exchange and correlation functional, on which the ACE potential was fitted.

#### 4.4.2 Interface Phase Diagram

To construct the interface phase diagram presented in Figure 3d, we considered all interface structures shown in Figure S8. All models are periodic in all three Cartesian directions, and thus contain two identical interfaces. Each slab of material, either FeO or Fe_3_O_4_, is at least 20 Å‐thick to minimize interactions with periodic images of the interfaces contained in the simulation cell. The equilibrium relative position of the two slabs is first determined by varying the position of the FeO slab relative to the Fe_3_O_4_ slab. After this first step, the separation distance between the two slabs is determined by minimizing the energy of the interface as a function of it. Finally, all interface models are fully relaxed while keeping the simulation cell fixed.

After this last static minimization step, we performed a short 10 ps MD run to anneal the interface structure and allow for its reconstruction. During this run, the spins of the Fe atoms are allowed to equilibrate by performing Monte–Carlo swaps between different spin species handled by the ACE Fe‐O potential (more details are given in Ref. [30] and in the previous section).

For each of the considered structure, the interface energy $\gamma_{\text{int.}}$ is evaluated as a function of the O chemical potential $\mu_{O}$ as:

(7) $$\begin{array}{cc} \gamma_{\text{int.}}\left(\mu_{O}\right)=\frac{1}{2\;S_{\text{int.}}} & \left[E_{\text{int.}}-\left[n_{\text{Fe}}^{\text{FeO}}\left(E_{\text{FeO}}-\mu_{O}\right)+n_{O}^{\text{FeO}}\;\mu_{O}\right]\right.\\ & \left.-\left[\frac{1}{3}\;n_{\text{Fe}}^{{\text{Fe}}_{3}O_{4}}\left(E_{{\text{Fe}}_{3}O_{4}}-4\;\mu_{O}\right)+n_{O}^{{\text{Fe}}_{3}O_{4}}\;\mu_{O}\right]\right], \end{array}$$

where the definition of the dependence of the energy of the two slabs is the same as for the surface formation energies presented above. $E_{\text{int.}}$ is the total energy of the periodic simulation cell containing two identical interfaces of surface area $S_{\text{int.}}$.

We also evaluate the adhesion energy $\gamma_{\text{adh.}}$ of these different interfaces (see Table S1), which are defined as:

(8) $$\gamma_{\text{adh.}}=\frac{E_{\text{int.}}-\sum_{i}E_{\text{slab}\;i}}{2\;S_{\text{int.}}},$$

where $E_{\text{int.}}$ is the total energy of the interface, $E_{\text{slab}\;i}$ are energies of the individual slabs in vacuum (here the FeO and the Fe_3_O_4_ slabs), and $S_{\text{int.}}$ is the area of the interface.

#### 4.4.3 Diffusion of Fe Interstitial Atoms to Surfaces

Additionally, we also studied segregation/diffusion of interstitial Fe atoms to surfaces of Fe_3_O_4_ using MD simulations. The snapshots presented in Figure 3e–g were extracted from a simulation performed at 1000 K during 2 ns. Along the MD simulation, the spins of the Fe atoms are allowed to equilibrate, as detailed before and in Ref. [30]. The slab model is periodic in the two in‐plane directions and contains two different terminations of the Fe_3_O_4_{111} surfaces: an O‐rich one on one side (corresponding to the $O_{1}$ termination), and an Fe‐rich one on the other side (in particular the ${\mathrm{Fe}}_{{\text{tet.}}_{1}}$ termination). We then insert 16 interstitial Fe atoms of random initial spin orientations in the middle of the slab along the direction of the plane normal. The simulation cell contains a total of 1104 atoms.

#### 4.4.4 Adatom Formation Energies and Potential Energy Surfaces

Formation energies of Fe and O adatoms are computed for the most probable surface facets of both Fe_3_O_4_ and FeO, i.e. ${\mathrm{Fe}}_{3}O_{4}{\left\{100\right\}}_{{\mathrm{FeO}}_{2}}$, ${\mathrm{Fe}}_{3}O_{4}{\left\{100\right\}}_{{\mathrm{FeO}}_{2}}^{\mathrm{SCV}}$, ${\mathrm{Fe}}_{3}O_{4}{\left\{111\right\}}_{O_{1}}$, ${\mathrm{Fe}}_{3}O_{4}{\left\{111\right\}}_{{\mathrm{Fe}}_{{\text{tet.}}_{1}}}$, FeO{100}, $\mathrm{FeO}{\left\{111\right\}}_{\mathrm{Fe}}$ and $\mathrm{FeO}{\left\{111\right\}}_{O}$. To avoid interactions between periodic images of the adatom in the in‐plane directions, the simulation cells used for these calculations contain a surface at least 15 Å‐long in each in‐plane direction.

The formation energy $E_{\text{ad.}}$ of the adatom on a given surface is defined as:

(9) $$E_{\text{ad.}}=E_{\text{surf.}\;+\;i}-\left(E_{\text{surf.}}+\mu_{i}\right),$$

where $E_{\text{surf.}\;+\;i}$ is the energy of the system containing two surfaces with one adatom on top of one of the two surfaces, $E_{\text{surf.}}$ is the energy of the system without the adatom, and $\mu_{i}$ is the chemical potential of the adatom species. Here, we take $\mu_{\text{Fe}}=\mu_{\text{Fe}}^{\text{BCC Fe}}$ and $\mu_{O}=1/2\mu_{O}^{O_{2}}$, i.e. Fe and O referenced to bulk BCC Fe and molecular O_2_, respectively.

We first evaluate the potential energy surface of the adatom on all considered surfaces, as presented in Figure S9, considering an Fe atom having both spin up and down, and an O atom. For this, the position of the adatom is relaxed in the direction normal to the surface plane, while all other atoms are kept fixed. The fully relaxed formation energies, presented in Table S2, are obtained by relaxing all atoms starting from the position of the adatom that minimizes the energy of the system.

#### 4.4.5 Diffusion Coefficients

As presented in the main text, we also evaluated the temperature dependent diffusion coefficient of interstitial Fe atoms ${\mathrm{Fe}}_{\text{int.}}$ using direct MD simulations. To do so, we set up a simulation cell of 1512 atoms with an additional single ${\mathrm{Fe}}_{\text{int.}}$ atom located at its most stable position (octahedral site with respect to the O face‐centred cubic sub‐lattice of Fe_3_O_4_). A MD simulation is then run, along which the mean squared displacement (MSD) of all Fe atoms contained in the simulation cell is recorded. The effective diffusion coefficient $D_{{\text{Fe}}_{\text{int.}}}$ of ${\mathrm{Fe}}_{\text{int.}}$ atoms is then computed at different temperatures T from the slope of the time‐dependent MSD at a given temperature T, i.e.:

(10) $$D_{{\text{Fe}}_{\text{int.}}}\left(T\right)=N_{\text{Fe}}\frac{{\text{MSD}}_{\text{Fe}}\left(T\right)}{6\;t},$$

where $N_{\text{Fe}}$ is the number of Fe atoms contained in the simulation cell, and t is the simulation time.

During the MD simulations, we noticed that the motion of ${\mathrm{Fe}}_{\text{int.}}$ atoms occurs through a ballistic mechanism that requires the spin of the moving ${\mathrm{Fe}}_{\text{int.}}$ atom to flip upon exchanging its position with a lattice Fe atom. Thus, the spins of the Fe atoms are dynamically equilibrated during the molecular dynamics simulation according to the methodology described above and in Ref. [30] to allow for such diffusion mechanism to occur.

After computing $D_{{\text{Fe}}_{\text{int.}}}$ at different temperatures, we fit its temperature dependence to the following equation:

(11) $$D_{{\text{Fe}}_{\text{int.}}}\left(T\right)=D_{{\text{Fe}}_{\text{int.}}}^{0}\exp\left(-\frac{E_{m}}{k_{B}T}\right),$$

where $D_{{\text{Fe}}_{\text{int.}}}^{0}$ is a prefactor, and $E_{m}$ is the activation energy for diffusion. We obtain $D_{{\text{Fe}}_{\text{int.}}}^{0}=5.16\times 10^{-8}$ $m^{2}$ $s^{-1}$ and $E_{m}=0.17$ eV. The characteristic diffusion length is then obtained as $L\left(T\right)=\sqrt{t\times D_{{\text{Fe}}_{\text{int.}}}\left(T\right)}$, with t the considered timescale, here 1 s.

## Conflicts of Interest

The authors declare no conflicts of interest.

## Supporting information




**Supporting File 1**: advs76583‐sup 0001 SuppMat.pdf.

**Supporting File 2**: advs76583‐sup‐0002‐VideoS1.mp4.

**Supporting File 3**: advs76583‐sup‐0003‐VideoS2.mp4.

**Supporting File 4**: advs76583‐sup‐0004‐VideoS3.mp4.

**Supporting File 5**: advs76583‐sup‐0005‐VideoS4.mp4.

## Data Availability

The data that support the findings of this study are available from the corresponding author upon reasonable request.
