# Supplementary material for: Cation Diffusion‐Mediated Displaced Reaction‐Transformation in Green Steelmaking
Source: Adv Sci (Weinh). 2026 Jul 16;13(56):e76583. doi: 10.1002/advs.76583 (PMC13373905; doi:10.1002/advs.76583)
Supplement: Supplementary file 1 [file ADVS-13-e76583-s002.pdf]

Cation Diffusion-mediated Displaced Reaction-transformation in Green Steelmaking

Guangyi Guo Paul Paciok Baptiste Bienvenu Longqi Bai Barak Ratzker Marc Heggen Xuyang Zhou* Dierk Raabe**

G. Guo, B. Bienvenu, L. Bai, B. Ratzker, X. Zhou, D. Raabe
Max Planck Institute for Sustainable Materials
Max-Planck-Straße 1, 40237 Düsseldorf, Germany
Email Address: b.bienvenu@mpi-susmat.de, x.zhou@mpi-susmat.de, d.raabe@mpi-susmat.de
P. Paciok, M. Heggen
Ernst Ruska-Centre for Microscopy and Spectroscopy with Electrons, Jülich Forschungszentrum
Wilhelm-Johnen-Straße, 52428 Jülich, Germany
L. Bai, X. Zhou
Center for Advancing Materials Performance from the Nanoscale (CAMP-Nano), State Key Laboratory for Mechanical Behavior of Materials, Xi'an Jiaotong University
710049 Xi'an, China

Keywords: *Hydrogen-based direct reduction, Cation diffusion, In situ transmission electron microscopy, Magnetite*

1 Supporting Information

1.1 Supplementary tables

Table S1. Computed properties of FeO/Fe_3O_4 interfaces. Stability range in $\Delta\mu_O$ and interface adhesion energy $\gamma_{adh.}$ for each of each of all considered interface configurations for the two {100} and {111} interfaces, defined by the terminations of the two FeO and Fe_3O_4 surfaces in contact.

Interface model	$\Delta\mu_O$ stability range	$\gamma_{adh.}$ (meV Å^{-2})
$Fe_3O_4\{100\}_{FeO_2}$ / $FeO\{100\}$	None	-142
$Fe_3O_4\{111\}_{Fe_{tet.1}}$ / $FeO\{111\}_O$	$-1.55\,eV < \Delta\mu_O \leq 0$	-123
$Fe_3O_4\{111\}_{Fe_{oct.2}}$ / $FeO\{111\}_O$	$\Delta\mu_O \leq -1.55\,eV$	-204
$Fe_3O_4\{111\}_{Fe_{oct.1}}$ / $FeO\{111\}_O$	None	-253
$Fe_3O_4\{111\}_{Fe_{tet.2}}$ / $FeO\{111\}_O$	None	-345
$Fe_3O_4\{111\}_{O_2}$ / $FeO\{111\}_{Fe_\downarrow}$	None	-412
$Fe_3O_4\{111\}_{O_2}$ / $FeO\{111\}_{Fe_\uparrow}$	None	-412
$Fe_3O_4\{111\}_{O_1}$ / $FeO\{111\}_{Fe_\downarrow}$	None	-426
$Fe_3O_4\{111\}_{O_1}$ / $FeO\{111\}_{Fe_\uparrow}$	None	-448

Table S2. Computed adatom formation energies on various surfaces of Fe_3O_4 and FeO. Adatom formation energies $E_{ad.}$ for O and Fe on surfaces of Fe_3O_4 and FeO discussed in the main text. Values for Fe adatoms are given for both spin up and spin down orientations, spin down being under brackets. For the evaluation of the adatom formation energies, Fe and O atoms are referenced to BCC Fe and molecular O_2, respectively.

Surface	Adatom	$E_{ad.}$ (eV)
$Fe_3O_4\{100\}_{FeO_2}$	Fe	+0.01 (+0.15)
	O	−0.38
$Fe_3O_4\{100\}^{SCV}_{FeO_2}$	Fe	−0.43 (−0.17)
	O	−0.18
$Fe_3O_4\{111\}_{O_1}$	Fe	−1.65 (−2.18)
	O	−0.84
$Fe_3O_4\{111\}_{Fe_{tet.1}}$	Fe	+0.74 (+0.44)
	O	−1.03

1.2 Supplementary figures

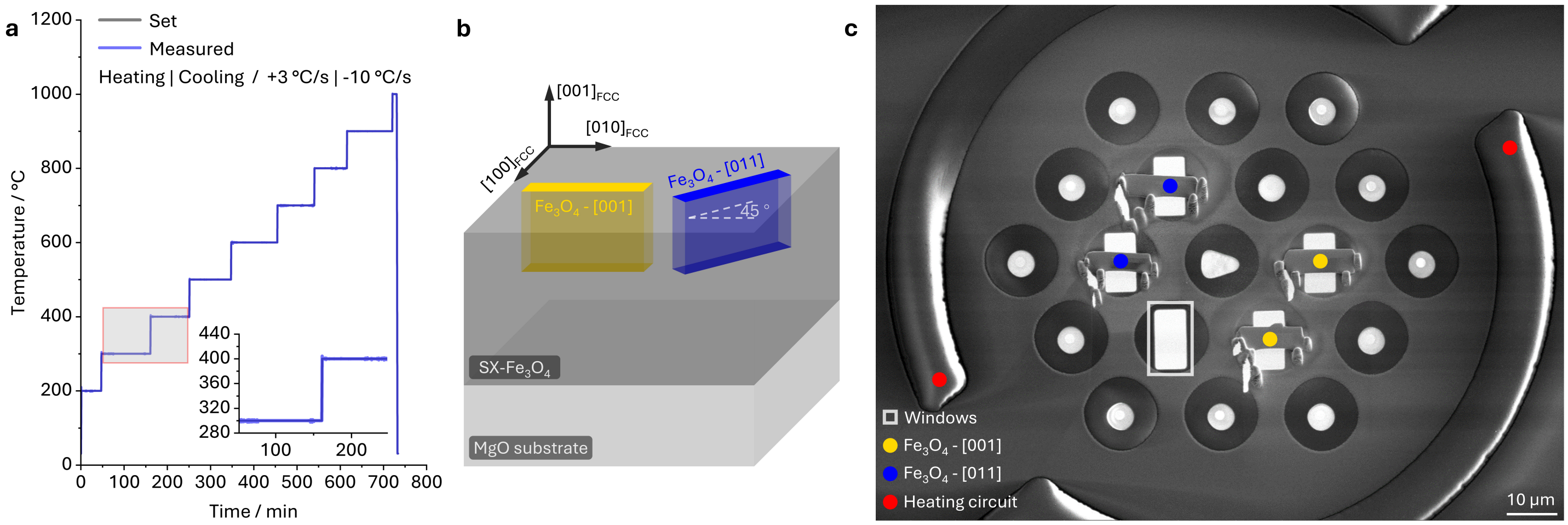

Figure S1. Reduction temperature profile and specimen placement on the heating chip. a) Reduction temperature profile used in the *in situ* reduction experiments. Specimens were heated stepwise from 200 °C to 1000 °C and then cooled down to room temperature. The heating and cooling rates were $+3\,°C\,s^{-1}$ and $-10\,°C\,s^{-1}$, respectively. As shown in the enlarged red inset, the measured temperature closely aligns with the set values, demonstrating temperature stability during the reduction process. b) A schematic illustrating the lift-out geometry used to prepare [001]- (yellow) and [011]- (blue) oriented SX lamellae. Here, FCC denotes face-centered cubic. c) A secondary-electron (SE) image acquired after specimen mounting showing the lift-out lamella positioned on the heating chip, along with additional windows (indicated by a white rectangle) that were intentionally milled to enable the thinning process during sample preparation. [001]- and [011]-oriented specimens are positioned above, indicated in yellow and blue, respectively. The surrounding spiral heating wires are marked with red spots.

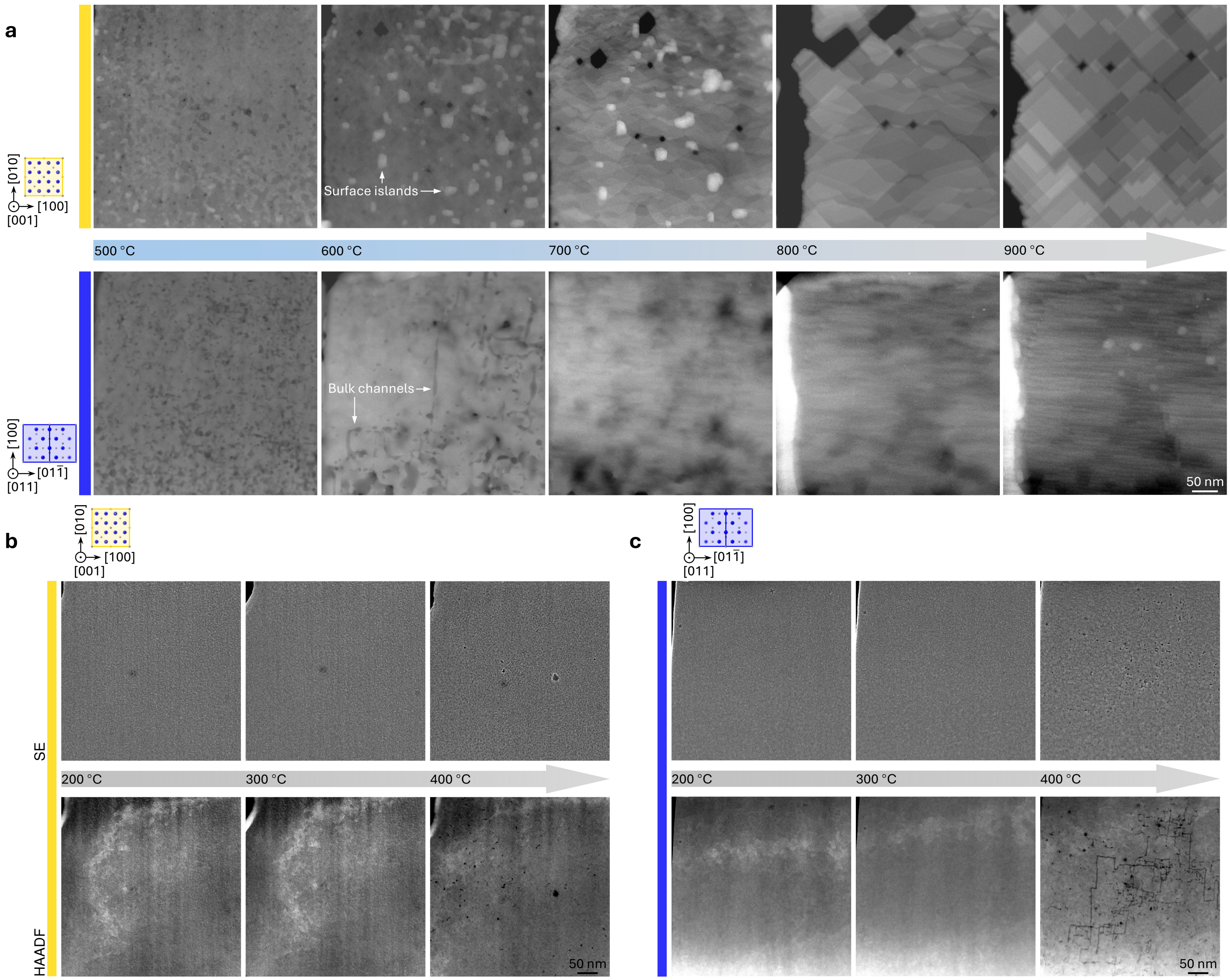

Figure S2. HAADF STEM and SE images showing the reduction evolution. a) Left icons give the projection and orientation information of Fe_3O_4. HAADF images of [001]- and [011]-oriented specimens reduced at various temperature stages are presented in the yellow and blue rows, respectively. Surface islands and bulk channels form at lower temperatures, while oriented terraces gradually appear as the temperature increases to 600 °C, as indicated by arrows. b,c) SE and HAADF STEM images showing the reduction evolution captured from 200 °C to 400 °C. Top icons give the projection and orientation information of (b) [001]-oriented and (c) [011]-oriented Fe_3O_4 specimen.

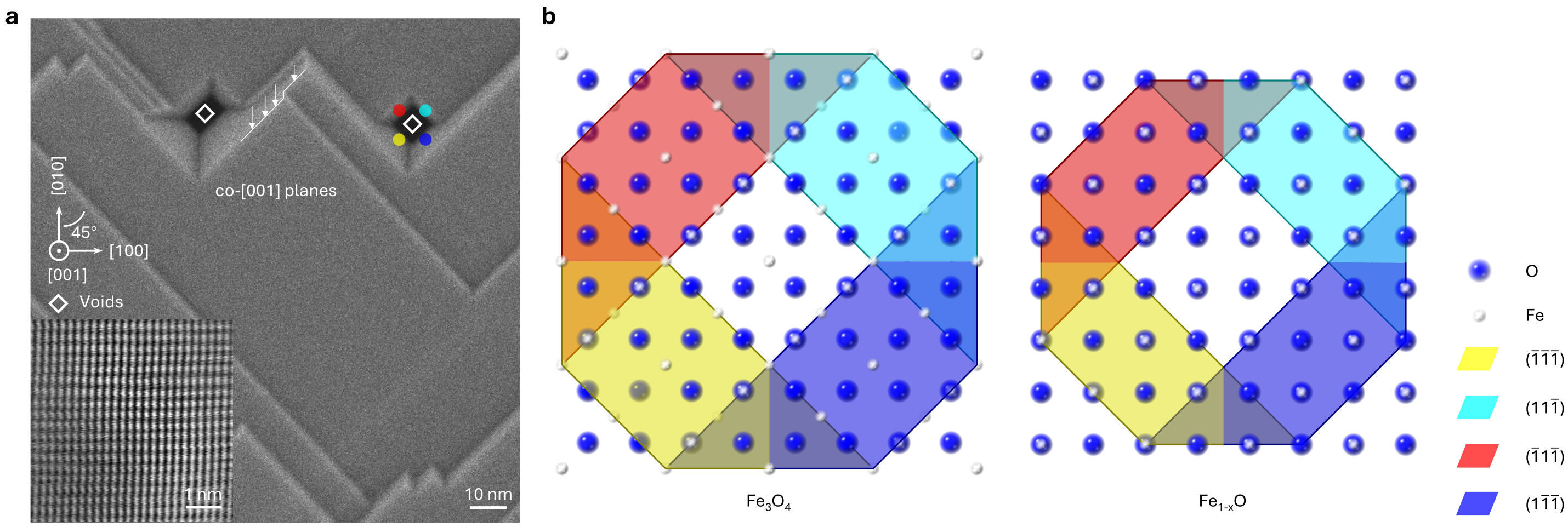

Figure S3. Crystallographic orientation of surface terraces. a) SE image showing the nanovoids enclosed by ledges and a 45° rotational relationship between ledges and [010] direction of initial Fe_3O_4 matrix. Ledges predominantly propagate along the {111} planes, as marked by white lines and arrows, and terminate upon encountering the large, low-surface-energy, passively formed co-[001] plane where the former ledges also stop. The high-resolution annular bright-field inset reveals the atomic structure of the surface ledges, which is consistent with the $Fe_{1-x}O$ structure when observed along the [001] direction. b) Schematic illustrating the formation of square frustum-shaped nanovoids due to the crystallography of terraces on the reduced specimen, four equivalent {111} planes shape the inverse trapezoid on the surface (Fe_3O_4 or $Fe_{1-x}O$). Blue and white spheres represent O and Fe atoms, respectively. Four equivalent {111} planes are distinguished by color.

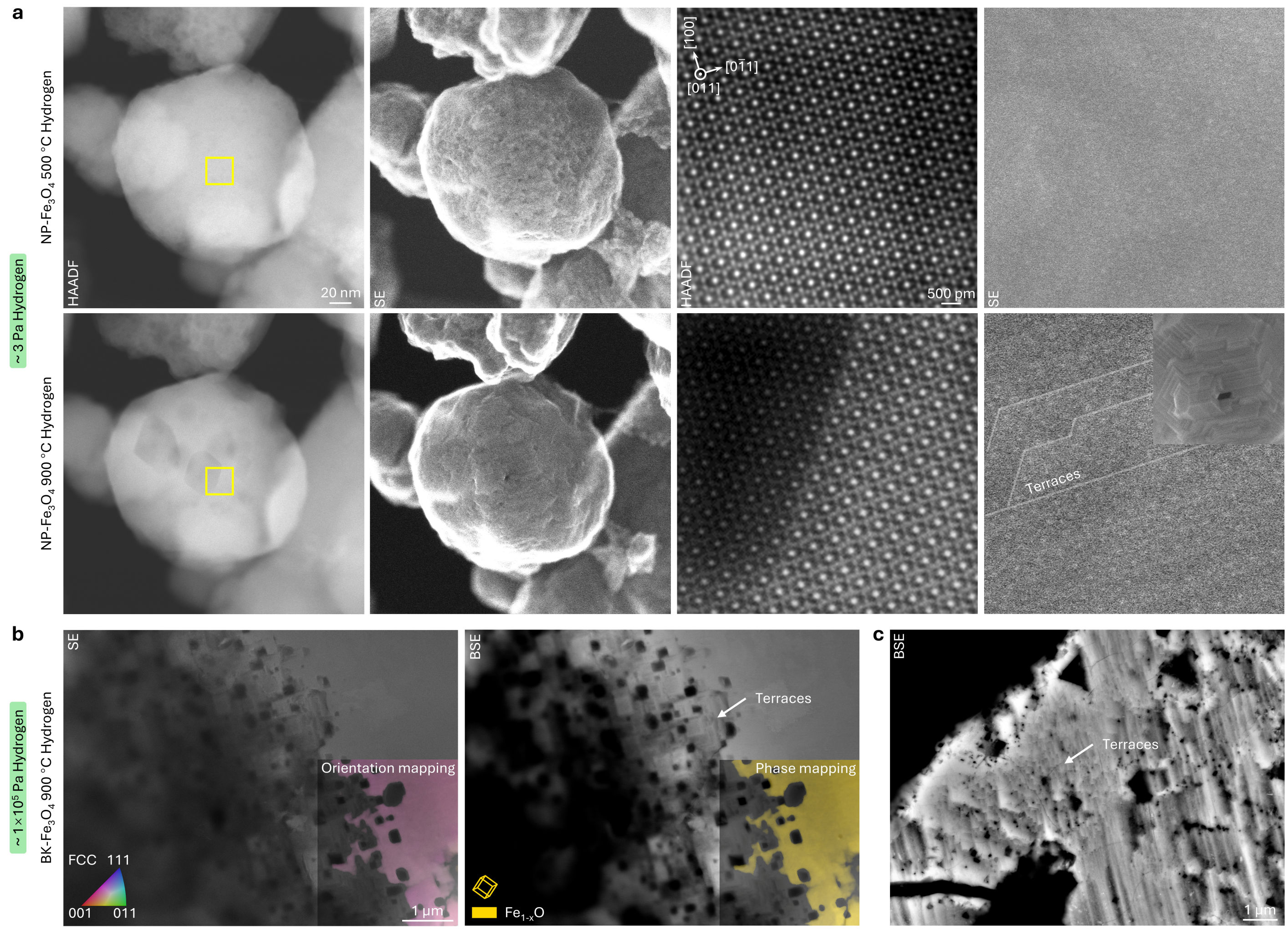

Figure S4. Surface terraces on Fe_3O_4 nanoparticles (NP-Fe_3O_4) and bulk ore (BK-Fe_3O_4) during hydrogen-based direct reduction. a) In situ HAADF and SE images of Fe_3O_4 nanoparticles reduced at 500 °C (first row) and 900 °C (second row), respectively. Acquired from the same domain, as indicated by yellow cubes, atomic terraces appear on the surface upon increasing the temperature to 900 °C, while the crystal structure of Fe_3O_4 remains preserved. The inset shows an overview image taken at lower magnification. b) Bulk Fe_3O_4 ore reduced at 900 °C, exhibiting terrace structures on the $Fe_{1-x}O$ surface (indicated by arrows). Insets and icons indicate crystallographic orientation and phase information obtained from EBSD. The EBSD signal is detected only in the uncovered region (indicated by color), as the resin-filled area diffuses backscattered electrons. c) Another region on the same powder sample as shown in (b), exhibiting similar surface terraces.

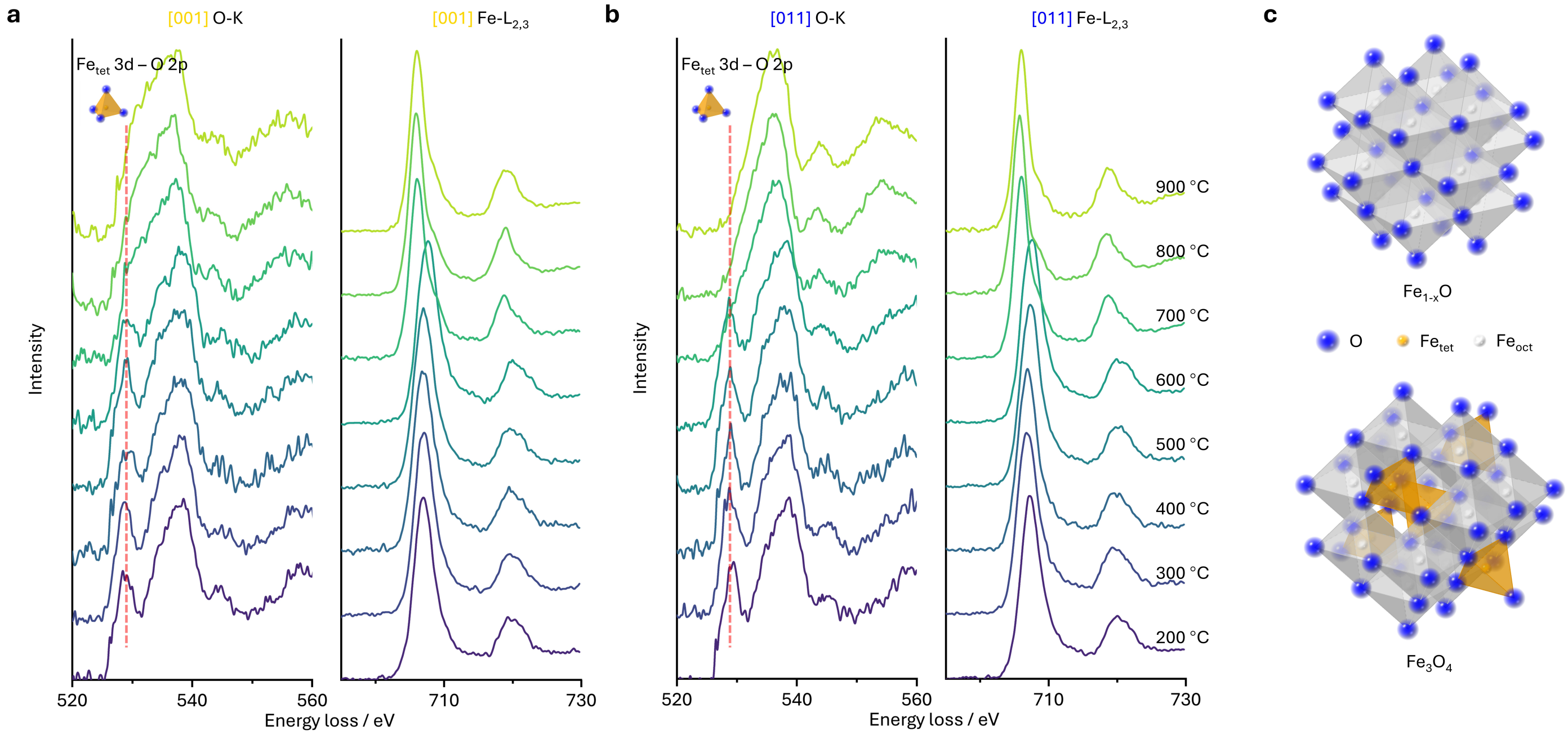

Figure S5. EELS acquired at defined temperatures, sectioned at the O K- and Fe $L_{2,3}$-edges. a) O-K edges (left) and Fe-$L_{2,3}$ (right) edges of the [001]-oriented specimen. b) O-K edges (left) and Fe-$L_{2,3}$ (right) edges of the [011]-oriented specimen. The red dashed lines and tetrahedral icons locate the position of prepeak prior to O-K, predominantly corresponding to the energy loss associated with Fe-O ionic bonds at tetrahedral site, where the $2p$ orbital of O interacts with the $3d$ orbital of Fe^{3+} (approximately 528 eV). When the reduction temperature exceeds 700 °C, the intensity of characteristic prepeak prior to O-K edge significantly diminishes on both [001]- and [011]-oriented specimens, confirming the reduction of tetrahedral Fe ions viz. the transformation from Fe_3O_4 to $Fe_{1-x}O$. c) Schematic of the tetrahedral and octahedral sites and crystal structure of Fe_3O_4 and $Fe_{1-x}O$.

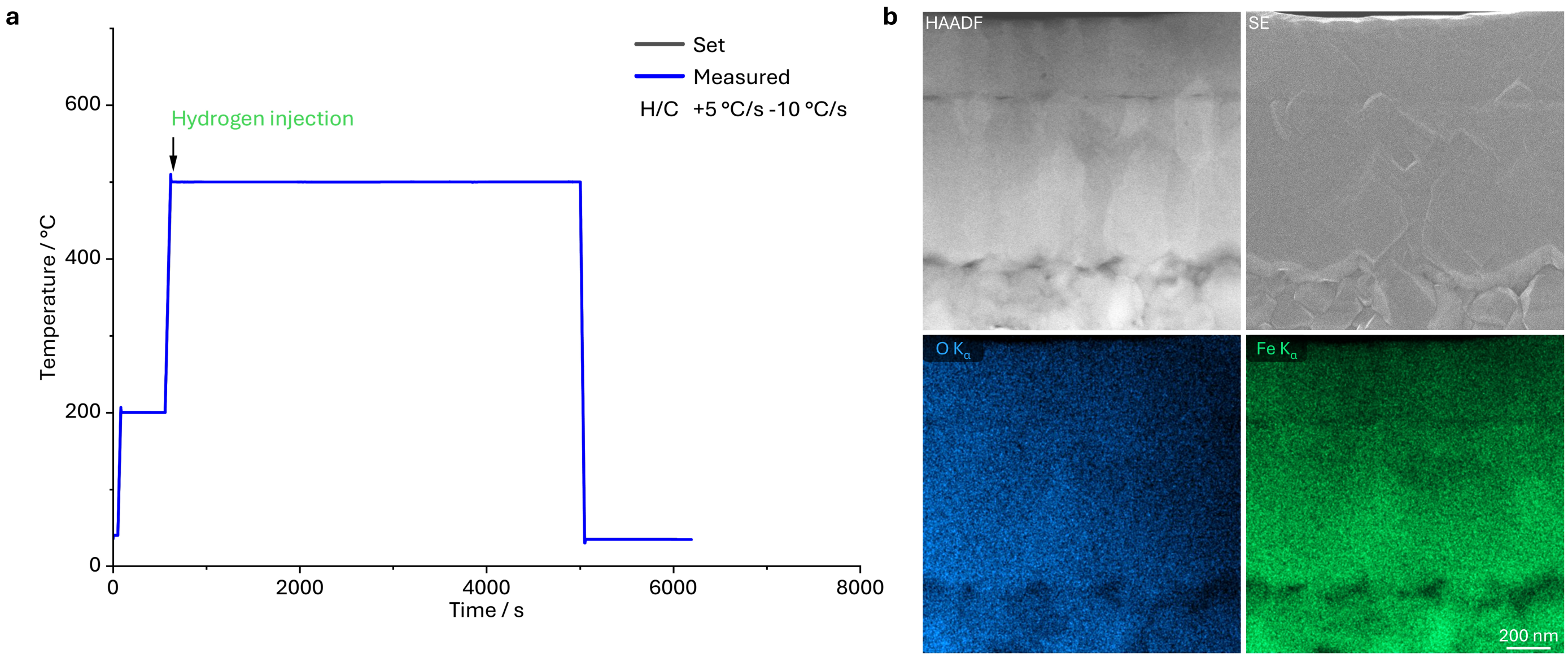

Figure S6. Depletion of Fe layer on multilayered Fe/Fe_3O_4 lamella after reducing at 500 °C. a) Reduction temperature profile used for multilayered lamella in the low temperature reduction group. Specimen were pre-heated to 200 °C to prevent possible organic carbon contamination and subsequently elevated to 500 °C. The heating and cooling rate are $+5\,^{\circ}\mathrm{C\,s^{-1}}$ and $-10\,^{\circ}\mathrm{C\,s^{-1}}$, respectively. b) Energy-dispersive X-ray spectroscopy mapping acquired after reduction showing the depletion of Fe layer on the reduced multilayered specimen. Simultaneous surface reconstruction shows up on both SX and PX domains.

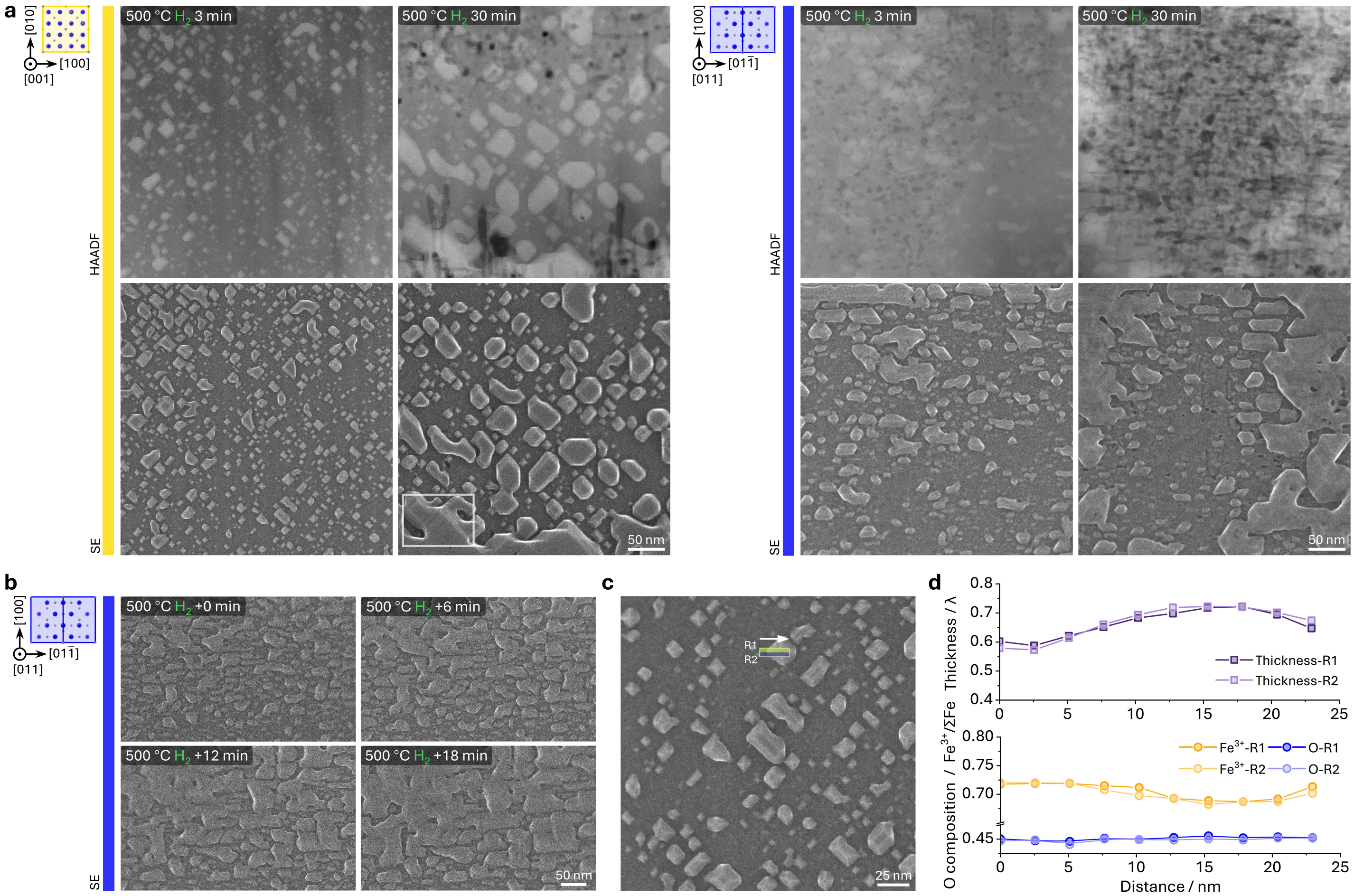

Figure S7. Evolution of reconstructed islands on the surface of SX Fe_3O_4 at 500 °C a) SE and HAADF images showing the formation and growth of truncated octahedral islands enclosed by {111} and {001} planes on [001]- and [011]-oriented surfaces, respectively. b) Time-series morphology of the [011]-oriented surface, showing progressive growth and eventual coalescence of surface islands, resulting in a reconstructed surface layer. c) SE image of a [001]-oriented specimen reduced for 3 min. One of the octahedral islands analyzed by EELS is marked with an arrow, at which R1 and R2 indicate two adjacent scanning pixel lines used for analysis. d) Quantitative results of line R1 and R2 showing the thickness and compositional evolution extracted from EELS spectrum.

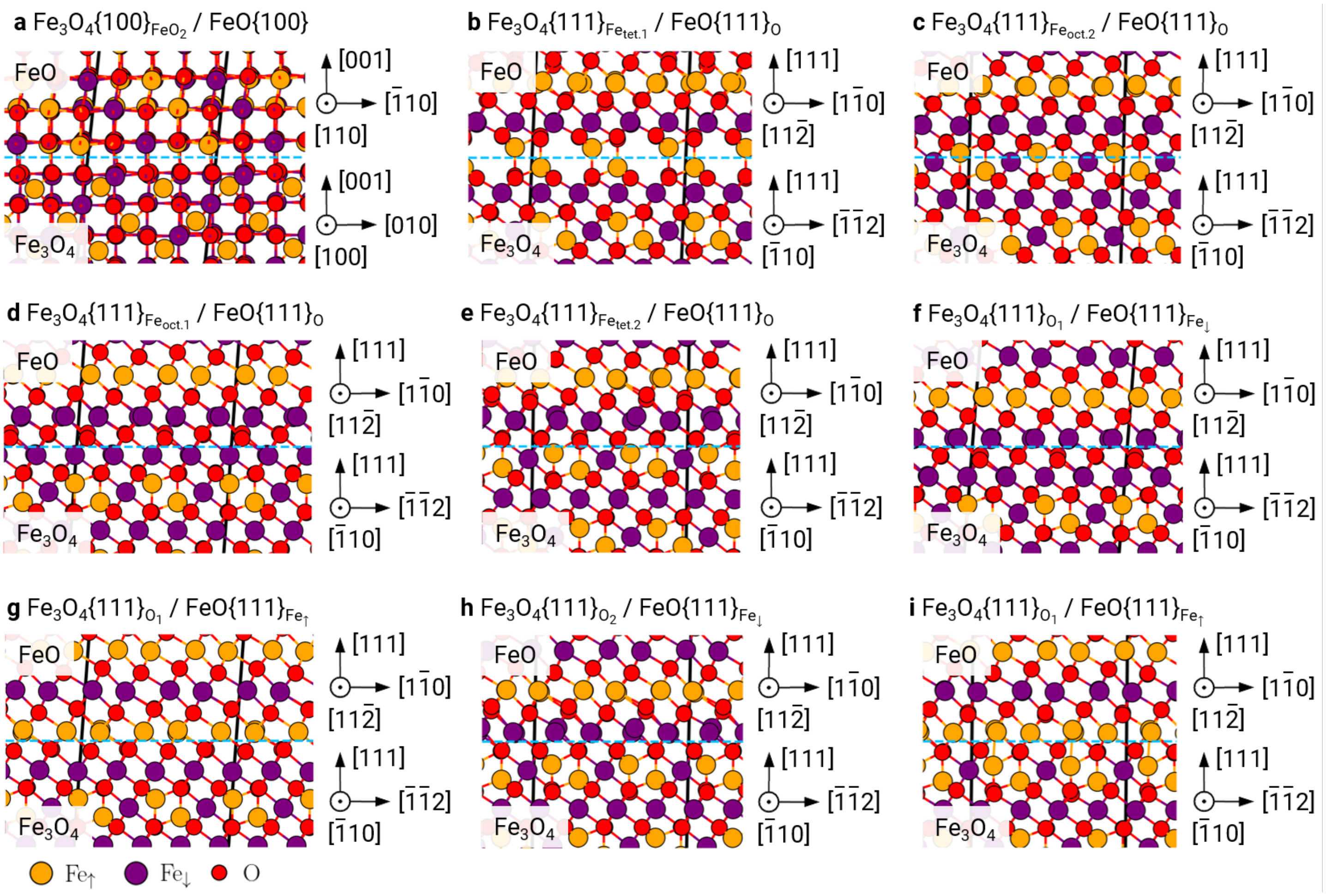

Figure S8. All considered FeO/Fe_3O_4 interface structures. All possible configurations of the interface are considered. Fe atoms are colored according to their spin orientation (orange for spin up and purple for spin down) and O atoms are in red. The interface plane is indicated in each panel by a light blue horizontal dashed line. For each interface configuration, both atomic positions and Fe spin orientations are relaxed.

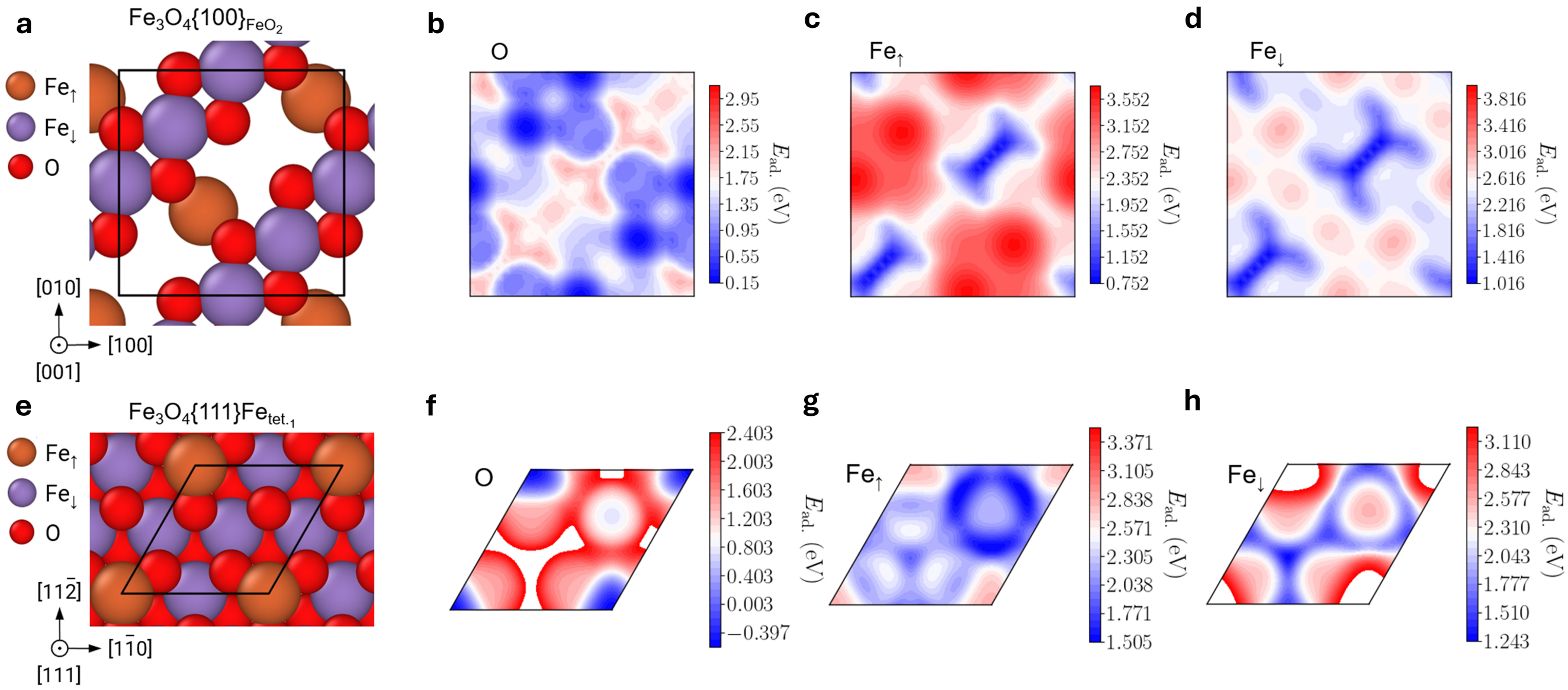

Figure S9. Adatom potential energy surface on the $Fe_3O_4\{111\}_{tet.1}$ surface and $Fe_3O_4\{100\}_{FeO_2}$ surface. Adatom formation energy as a function of its position on the $Fe_3O_4\{111\}_{tet.1}$ surface, whose structure is shown in (a), considering (b) O, (c) $Fe_\uparrow$ and (d) $Fe_\downarrow$ atoms. The potential energy surface is evaluated by relaxing the position of the adatom in the direction normal to the surface while keeping all other atoms fixed. Fully relaxed adatom formation energies are presented in Table S2 (Supporting Information) for different surfaces. Identical calculation to the $Fe_3O_4\{100\}_{FeO_2}$ surface are shown in (e), considering (f) O, (g) $Fe_\uparrow$ and (h) $Fe_\downarrow$ atoms.

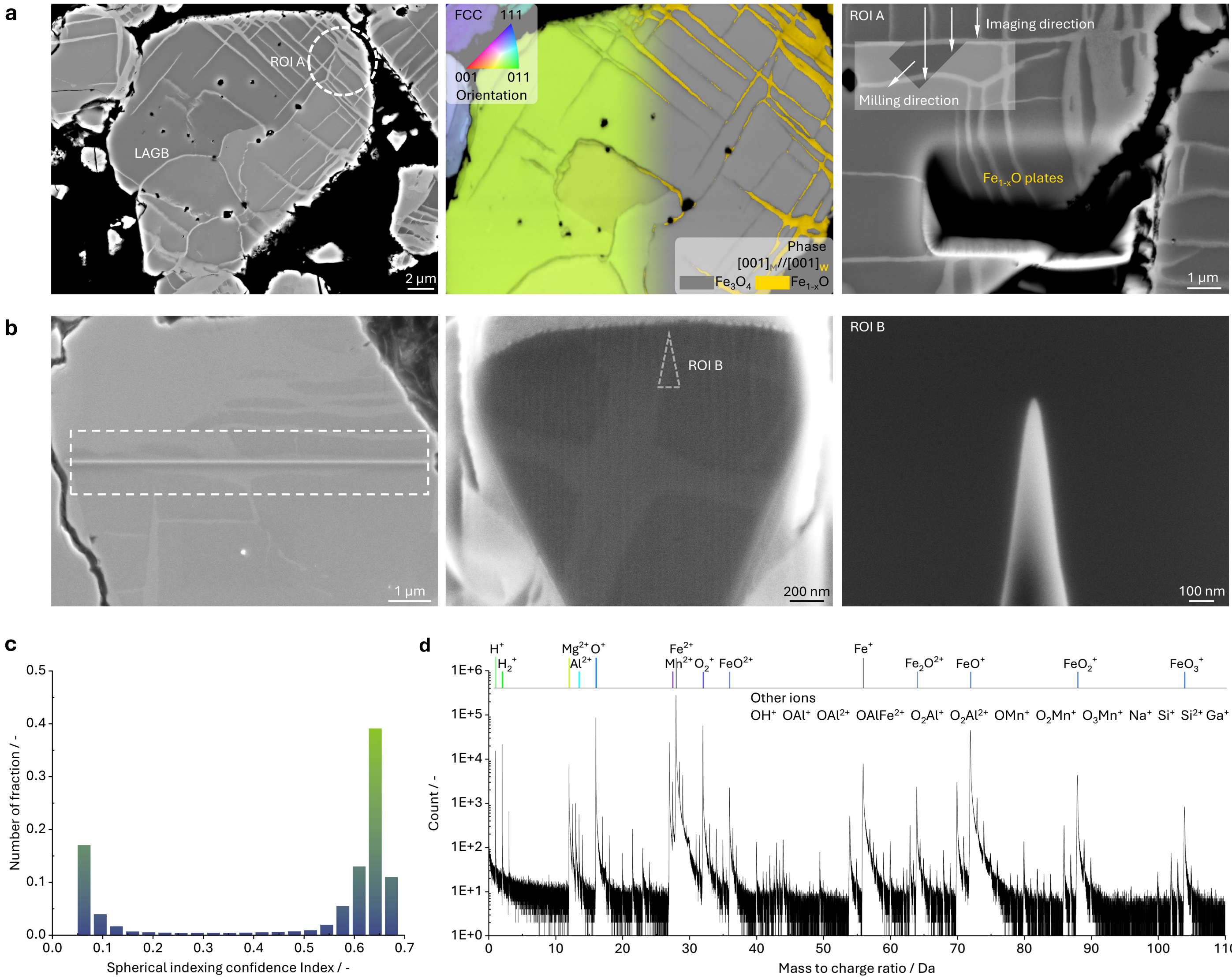

Figure S10. Three-dimensional plate-like distribution of $Fe_{1-x}O$ within Fe_3O_4. a) Fe_3O_4 ore reduced at 900 °C for 0 s, showing a plate-like distribution of $Fe_{1-x}O$. A low-angle grain boundary (LAGB) is preferentially reduced to $Fe_{1-x}O$. The corss-section BSE image showing a 45° inclined milling process was performed at region of interest (ROI) A to expose the three-dimensional structure of $Fe_{1-x}O$ plates beneath the polished surface. b) Site-specific lift-out process for specimen preparation for atom probe tomography (APT). The dashed rectangle marks a triangular prism encompassing the phase boundary between Fe_3O_4 and $Fe_{1-x}O$. The dashed triangle within the prism, labeled with ROI B, indicates the position of the APT tip including phase boundary. c) Confidence Index (CI) distribution of data processed using spherical indexing techniques. A minimum CI threshold of 0.5 is applied to ensure reliable phase and orientation mapping results. d) Mass spectrum acquired during the evaporation of the APT tip, ranging from 0 to 110 Da (relative atomic mass charge ratio). In addition to the major Fe and oxide species, the rest minor complex ions are listed separately.
